# Supplementary material for: ReIMAGINE Prostate Cancer Screening Study: protocol for a single-centre feasibility study inviting men for prostate cancer screening using MRI
Source: BMJ Open. 2021 Sep 30;11(9):e048144. doi: 10.1136/bmjopen-2020-048144 (PMC8487192; doi:10.1136/bmjopen-2020-048144)
Supplement: Supplementary data [file bmjopen-2020-048144supp001.pdf]

Appendix I: Amendments made to the ReIMAGINE Prostate Cancer Screening Study Protocol

Two non-substantial amendments were made to the study protocol during the duration of the study:

Non-substantial amendment 1

The first non-substantial amendment was submitted for review in June 2020. Review by the study sponsor (UCL) determined this was a category C amendment that did not require study wide review, with notification to UK Health Research Authority only. The purpose of the amendment was modification of study documents (Participant Information Sheet, PIS) to navigate participants to the study privacy notice which informed participants about the information being collected and its intended use, a General Data Protection Regulation (GDPR) requirement. At this time, we incorporated minor updates to the study protocol (expansion on what categories of aggregate data we intend to collect from PIC sites / GP practices) and the study consent form (mention of the privacy notice) to correct administrative omissions. The amendment was implemented at site in July 2020.

Non-substantial amendment 2

The second non-substantial amendment followed a temporary closure of recruitment to the study enforced by the study sponsor and NHS site in response to the COVID-19 pandemic (March 2020). The study team consulted with the Patient and Public Involvement (PPI) sub-committee on what modifications we may need to make to documentation or study design so to safely re-open to recruitment and provide assurance to potential new participants that COVID-19 concerns had been considered. This was submitted in July 2020, once again categorised as category C, and implemented in August 2020 when approval to reopen recruitment was granted by the study sponsor.

In response to the COVID-19 pandemic, the PIS was amended to reflect the change in travel allowance/arrangements made by the study team. All participants were offered the option of a private return taxi for their study visit, or reasonable travel allowance greater than the original £30. The study invitation letter (sent by GPs) was also updated to note the same change and navigate men to a new one-page information sheet in relation to study safeguards during the COVID-19 pandemic, the content of which was largely driven by the PPI committee.
