# Supplementary material for: ReIMAGINE Prostate Cancer Screening Study: protocol for a single-centre feasibility study inviting men for prostate cancer screening using MRI
Source: BMJ Open. 2021 Sep 30;11(9):e048144. doi: 10.1136/bmjopen-2020-048144 (PMC8487192; doi:10.1136/bmjopen-2020-048144)
Supplement: Supplementary data [file bmjopen-2020-048144supp002.pdf]

Appendix II: MRI protocol

All studies will be performed with the same protocol on a single 3 T scanner (Achieva®, Philips Healthcare, Netherlands) using a 32-channel pelvic-phased array coil and a bi-parametric acquisition protocol. Briefly, sequences will include an axial turbo spin echo and axial diffusion weighted imaging using a high b value at 2000 s mm–2. Exemplar sequence parameters are illustrated in the table below. Total protocol acquisition time <10 minutes.

Clinical bi-parametric MRI at 3T

Parameter	T_2 axial	DWI b 2000 s mm^{-2}
**Repetition time (ms)**	5407	2000
**Echo time (ms)**	100	78
**Flip angle (degree)**	90	90
**Orientation**	Axial	Axial
**Slice thickness (mm)**	3	5
**Matrix size**	300 × 290	168 × 169
**Field of view (mm)**	180	220
**Fat suppression**	No	SPIR
**Time for scan (min)**	05:13	03:40

The above images will be prospectively reported by radiologists to ascribe a positive/negative screen result as described in the ReIMAGINE Prostate Cancer Screening study protocol. Reports will be generated in UCLH EPIC using a study specific reporting template.

At the same sitting, patients will undergo exploratory imaging for acquisition of imaging to derive Luminal Water Fraction (LWF) maps. Imaging will be performed with the same scanner and coil setup. An 8-echo multi-echo spin echo sequence will be employed to provide axial imaging of the prostate. Exemplar sequence parameters are illustrated in the table below. Total exploratory imaging protocol time < 10 minutes.

Exploratory LWF MRI at 3T

**Parameter**	**LWF Imaging**
**Repetition time (ms)**	8956
**Echo time/spacing (ms)**	31.25/31.25
**Flip angle (degree)**	90
**Orientation**	Axial
**Slice thickness (mm)**	4
**Matrix size**	300 × 290
**Field of view (mm)**	180
**Fat suppression**	No
**Time for scan (min)**	05:13

Positive/negative screen status/quantitative analysis and concordance with the bi-parametric MRI will be performed. Reports will not be made available to the clinical team.
