# Supplementary material for: ReIMAGINE Prostate Cancer Screening Study: protocol for a single-centre feasibility study inviting men for prostate cancer screening using MRI
Source: BMJ Open. 2021 Sep 30;11(9):e048144. doi: 10.1136/bmjopen-2020-048144 (PMC8487192; doi:10.1136/bmjopen-2020-048144)
Supplement: Supplementary data [file bmjopen-2020-048144supp003.pdf]

Appendix III – The ReIMAGINE Screening Study Group

**Trial Sponsor:**	University College London (UCL)
**Trial Coordination:**	UCL Urology, Research Department of Targeted Intervention, Division of Surgery and Interventional Science
**Funders:**	The Medical Research Council, U.K. (MRC), grant number MR/R014043/1 and Cancer Research U.K. (CRUK)

Trial Management Group

**Caroline M Moore**	Co-Chief Investigator, Urologist - UCLH / UCL
**Mark Emberton**	Co-Chief Investigator, Urologist - UCLH / UCL
**Shonit Punwani**	Co-Investigator, Radiologist - UCLH / UCL
**Neil McCartan**	Clinical Project Management – UCL
**Louise Brown**	Co-Investigator – UCL MRC Clinical Trials Unit
**Mieke Van Hemelrijck**	Co-Investigator, Cancer Epidemiologist - KCL
**Ton Coolen**	Co-Investigator, Applied Mathematics - London Institute for Mathematical Sciences
**Aida Santaollala**	Cancer Epidemiology Group – KCL
**Teresa Marsden**	Clinical Research Fellow, Urology – UCLH / UCL
**Harbir Sidhu**	Consultant Radiologist – UCLH
**Douglas Kopcke**	Honorary Consultant Radiologist - UCLH
**Giorgio Brembilla**	Clinical Research Fellow, Radiology – UCLH / UCL
**Francesco Giganti**	Clinical Research Fellow, Radiology – UCLH / UCL

ReIMAGINE Screening Study Group

**Joanna Hadley**	Clinical Trial Practitioner – UCL
**Fatima Akbar**	Clinical Trial Practitioner - UCL
**Ged Corbett**	Project Manager – UCL TRO
**Manuel Rodriguez-Justo**	Pathologist – UCLH / UCL
**Elizabeth Isaac**	Imaging Trials Unit - UCLH
**Steve Tuck**	ReIMAGINE Consortium Patient Representative
**Saran Green**	Patient and Public Involvement - KCL
**Charlotte Moss**	Data Management - KCL
**Anna J Haire**	Data Management - KCL

**Teresita Beeston**	Imaging Trials Unit - UCLH
**Joey Clement**	Imaging Trials Unit - UCLH
**Fiona Gong**	Centre for Medical Imaging – UCL
**Lucy Casselton**	Centre for Medical Imaging – UCL
**Anna Wingate**	Laboratory Manager – UCL
**Katerina Soteriou**	Imaging Trials Unit - UCLH
**Charlotte Bevan**	Biological Research Committee - ReIMAGINE
**Paul Boutros**	Biological Research Committee - ReIMAGINE
**Andrew Feber**	Biological Research Committee - ReIMAGINE
**Hayley Whitaker**	Biological Research Committee - ReIMAGINE
**Caroline Dive**	Scientific Advisory Board - ReIMAGINE
**Eytan Domany**	Scientific Advisory Board - ReIMAGINE
**Malcolm Mason**	Scientific Advisory Board - ReIMAGINE
**Anwar Padhani**	Scientific Advisory Board - ReIMAGINE
**Eric Aboagye**	Steering Committee - ReIMAGINE
**Richard Kaplan**	Steering Committee - ReIMAGINE
**Chris Parker**	Steering Committee - ReIMAGINE
**Peter Parker**	Steering Committee - ReIMAGINE
