# Supplementary material for: ReIMAGINE Prostate Cancer Screening Study: protocol for a single-centre feasibility study inviting men for prostate cancer screening using MRI
Source: BMJ Open. 2021 Sep 30;11(9):e048144. doi: 10.1136/bmjopen-2020-048144 (PMC8487192; doi:10.1136/bmjopen-2020-048144)
Supplement: Author's manuscript [file bmjopen-2020-048144.draft_revisions.pdf]

BMJ Open

BMJ Open is committed to open peer review. As part of this commitment we make the peer review history of every article we publish publicly available.

When an article is published we post the peer reviewers' comments and the authors' responses online. We also post the versions of the paper that were used during peer review. These are the versions that the peer review comments apply to.

The versions of the paper that follow are the versions that were submitted during the peer review process. They are not the versions of record or the final published versions. They should not be cited or distributed as the published version of this manuscript.

BMJ Open is an open access journal and the full, final, typeset and author-corrected version of record of the manuscript is available on our site with no access controls, subscription charges or pay-per-view fees (http://bmjopen.bmj.com).

If you have any questions on BMJ Open's open peer review process please email info.bmjopen@bmj.com

BMJ Open

ReIMAGINE Prostate Cancer Screening Study – A single centre feasibility study inviting men for prostate cancer screening using MRI.

Journal:	*BMJ Open*
Manuscript ID	bmjopen-2020-048144
Article Type:	Protocol
Date Submitted by the Author:	24-Dec-2020
Complete List of Authors:	Marsden, Teresa; University College London, Department of Urology; University College London Hospitals NHS Foundation Trust, Department of Urology<br>Lomas, Derek J; Mayo Clinic, Department of Urology<br>McCartan, Neil; University College London, Department of Urology; University College London Hospitals NHS Foundation Trust, Department of Urology<br>Hadley, Joanna; University College London, Department of Urology; University College London Hospitals NHS Foundation Trust, Department of Urology<br>Tuck, Steve<br>Brown, Louise; University College London<br>Haire, Anna ; King's College London<br>Moss, Charlotte; King's College London, Translational Oncology and Urology Research<br>Green, Saran<br>Van Hemelrijck, Mieke; Kings College London, Translational Oncology & Urology Offices<br>Coolen, Ton; London Institute for Mathematical Sciences<br>Santaolalla, Aida ; King's College London, Translational Oncology and Urology Research<br>Isaac, Elizabeth; University College London<br>Brembilla, Giorgio; University College London<br>Kopcke, Douglas; University College London Hospitals NHS Foundation Trust, Department of Radiology<br>Giganti, Francesco; University College London, Department of Radiology; University College London Hospitals NHS Foundation Trust, Department of Radiology<br>Sidhu, Harbir; University College London<br>Punwani, Shonit; University College London Medical School, Centre for Medical Imaging<br>Emberton, Mark; University College London, Division of Surgery and Interventional Sciences; University College London Hospitals NHS Foundation Trust, Department of Urology<br>Moore, Caroline; University College London, Division of Surgery and Interventional Sciences
Keywords:	Magnetic resonance imaging < RADIOLOGY & IMAGING, Prostate disease < UROLOGY, Urological tumours < ONCOLOGY, Epidemiology <

	ONCOLOGY

SCHOLARONE™
Manuscripts

I, the Submitting Author has the right to grant and does grant on behalf of all authors of the Work (as defined in the below author licence), an exclusive licence and/or a non-exclusive licence for contributions from authors who are: i) UK Crown employees; ii) where BMJ has agreed a CC-BY licence shall apply, and/or iii) in accordance with the terms applicable for US Federal Government officers or employees acting as part of their official duties; on a worldwide, perpetual, irrevocable, royalty-free basis to BMJ Publishing Group Ltd ("BMJ") its licensees and where the relevant Journal is co-owned by BMJ to the co-owners of the Journal, to publish the Work in this journal and any other BMJ products and to exploit all rights, as set out in our licence.

The Submitting Author accepts and understands that any supply made under these terms is made by BMJ to the Submitting Author unless you are acting as an employee on behalf of your employer or a postgraduate student of an affiliated institution which is paying any applicable article publishing charge ("APC") for Open Access articles. Where the Submitting Author wishes to make the Work available on an Open Access basis (and intends to pay the relevant APC), the terms of reuse of such Open Access shall be governed by a Creative Commons licence – details of these licences and which Creative Commons licence will apply to this Work are set out in our licence referred to above.

Other than as permitted in any relevant BMJ Author's Self Archiving Policies, I confirm this Work has not been accepted for publication elsewhere, is not being considered for publication elsewhere and does not duplicate material already published. I confirm all authors consent to publication of this Work and authorise the granting of this licence.

For peer review only

ReIMAGINE Prostate Cancer Screening Study – A single centre feasibility study inviting men for prostate cancer screening using MRI.

Teresa Marsden [1,2], Derek J Lomas [3], Neil McCartan [1,2], Joanna Hadley [1,2], Steve Tuck [4], Louise Brown [5], Anna J Haire [6], Charlotte L Moss [6], Saran Green [6], Mieke Van Hemelrijck [6], Ton Coolen [7], Aida Santaolalla [8], Elizabeth Isaac [9], Giorgio Brembilla [9], Douglas Kopcke [10], Francesco Giganti [1,10], Harbir Sidhu [9], Shonit Punwani [9], Mark Emberton* [1,2], Caroline M Moore* [1,2] on behalf of the ReIMAGINE Study Group.

* Joint senior authors

[1] UCL Division of Surgical & Interventional Sciences, University College London, London, UK

[2] Department of Urology, University College London Hospitals NHS Foundation Trust, London, UK

[3] Department of Urology, Mayo Clinic, Rochester, Minnesota, USA

[4] ReIMAGINE Consortium Patient Representative

[5] MRC Clinical Trials Unit, University College London, London, UK

[6] School of Cancer and Pharmaceutical Sciences, Kings College London, London, UK

[7] London Institute for Mathematical Sciences, 35A South Street, Mayfair, London W1K 2XF

[8] Cancer Epidemiology Group, Division of Cancer Studies, King's College London School of Medicine, London, UK

[9] Centre for Medical Imaging, University College London, London, UK

[10] Department of Radiology, University College London Hospitals NHS Foundation Trust, London, UK

Contributors The study concept and design was conceived by CMM, ME, SP, LB, NMC, AJH, CLM and MVH. JH, TM EI, GB, DK, FG, HS and SP will conduct screening and data collection. Analysis will be performed by LB, MVH, TC, AS, SP and CMM. CMM, NMC, DJL and TM prepared the first draft of the manuscript. All authors provided edits and critiqued the manuscript for intellectual content.

Correspondence to:

Ms Teresa Marsden BMedSci (Hons), BMBS, MRCS (Eng)

UCL Division of Surgical & Interventional Sciences

3rd Floor, Charles Bell House

43-45 Foley Street

London

W1W 7TS

teresa.marsden@ucl.ac.uk

Running title: ReIMAGINE Prostate Cancer Screening

Total word count: 3124

Keywords: Magnetic Resonance Imaging, mpMRI; Prostate Cancer; Screening; Prostate Specific Antigen, PSA.

Abbreviations: Electronic Case Report Form, eCRF; European Union, EU; General Practitioner (GP); multi-parametric magnetic resonance imaging, mp-MRI; magnetic resonance imaging, MRI; material transfer agreement, MTA; multiparametric magnetic resonance imaging, mpMRI; National Health Service, NHS; prostate cancer, PCa; prostate specific antigen, PSA; Research Ethics Committee, REC; Standard Operating Procedure, SOP; Transrectal Ultrasound, TRUS; University College London, UCL.

For peer review only

Abstract

Introduction: The primary objective of the ReIMAGINE prostate cancer screening study is to explore the uptake of an invitation to prostate cancer screening using magnetic resonance imaging (MRI).

Methods and Analysis: The ReIMAGINE prostate cancer screening study is a prospective single center feasibility study (registration number NCT04063566). Eligible men aged 50 to 75 years with no prior prostate cancer diagnosis or treatment will be identified through general practitioner practices and randomly selected for invitation. Those invited will be offered an MRI scan and prostate specific antigen (PSA) blood test. The screening MRI scan consists of T2-weighted, diffusion-weighted and research specific sequences, without the use of intravenous contrast agents. Men who screen positive on either MRI or PSA density will be recommended to have standard of care (National Health Service) tests for prostate cancer assessment, which includes multi-paremetric MRI. The study will assess the acceptability of an MRI based prostate screening assessment and the prevalence of cancer detected in MRI screened men. Summary statistics will be used to explore baseline characteristics in relation to acceptance rates and prevalence of cancer.

Ethics and Dissemination: ReIMAGINE prostate cancer screening is a single site screening study to assess the feasibility of MRI as a screening tool for prostate cancer. Ethical Approval was granted by London – Stanmore Research Ethics Committee Heath Research Authority (reference 19/LO/1129). Study results will be published in peer reviewed journals after completion of data analysis and used to inform the design of a multi-centre screening study in the UK.

STRENGTHS AND LIMITATIONS OF THE STUDY

- Few studies have evaluated the use of MRI as a population screening test for prostate cancer.
- Recruitment to ReIMAGINE Screening mirrors formal UK screening programmes of invitation via GP practice alone.
- The study will assess the acceptability of MRI as a screening tool, the prevalence of MRI defined suspicious lesions and any subsequent prostate cancer detected as a result of their screening invitation.
- The study does not exclude men with a history of PSA testing, previous negative prostate biopsies or prostate MRIs and therefore provides an heterogenous population representative of the general population.

For peer review only

- Participation identification centres (GP practices) are limited to the London area limiting generalizability of findings.

INTRODUCTION

Prostate cancer is the most common cancer in men and second most common cause of male cancer-related death in the UK.[1] Cancer screening seeks to detect disease before it becomes clinically apparent to allow effective treatment. The UK has no prostate cancer screening programme, but men can ask for a prostate specific antigen (PSA) test via their general practitioner (GP), and men with a raised PSA or an abnormal digital rectal examination (DRE) are encouraged to undergo hospital-based testing.

Several large randomized population-based studies have been conducted to determine the long-term effect of a prostate cancer screening programme on overall and prostate cancer specific mortality. The Prostate, Lung, Colorectal and Ovarian (PLCO) screening study randomized over 76,000 men to annual PSA screening or usual care.[2] It showed no difference in prostate cancer mortality in the screened and control arms but has been criticized because half of the control arm had a PSA test during the study. The European Randomised Screening for Prostate Cancer (ERSPC) study provides 16-year follow up data for 162,389 men (age 55–69), radomised to PSA screening and control groups.[3, 4] Prostate cancer mortality was lower in the screening arm and relative to 13 year data, both the number needed to screen and diagnose fell suggesting a benefit to screening in the longer term. However, the number needed to diagnose remains high at 18, suggesting considerable overdiagnosis. In the UK Cluster randomised trial of PSA testing for prostate cancer (CAP) of 419,582 men, using a single PSA test, followed by a standard transrectal biopsy if the PSA was between 3 and 19.9 ng/ml, there was no difference in prostate cancer mortality in the PSA screened and control arms.[5] While currently available screening methods may reduce prostate cancer mortality, they are associated with an increased risk of over-diagnosis, over-treatment and biopsy complications.

Magnetic Resonance Imaging (MRI) performs better than standard biopsy in men with a clinical suspicion of prostate cancer based on a raised PSA or abnormal DRE. The UK PROMIS study compared multiparametric MRI (mpMRI) and 12-core TRUS biopsy with 5mm transperineal mapping biopsy as a gold-standard comparator. Almost twice as many clinically significant cancers were detected on mpMRI as on systematic sampling.[6] The international PRECISION study randomised men to either standard TRUS biopsy or an mpMRI with targeted biopsy alone for those men with a lesion scoring ≥3 on

For peer review only - http://bmjopen.bmj.com/site/about/guidelines.xhtml

mpMRI.[7] The MRI-based strategy resulted in increased diagnosis of clinically significant prostate cancer (38% vs 26%), with a reduction in detection of indolent disease (9% vs 22%) with 28% of men avoiding biopsy in the MRI arm. The MRI First and 4M studies have confirmed that mpMRI and targeted biopsy do not reduce the detection of clinically significant prostate cancer, but could allow up to 50% of men to avoid biopsy, and significantly reduce over-detection.[8, 9]

The next challenge is to see if the benefits of MRI in men with a suspicion of prostate cancer can be translated to the screening setting.

STUDY INFORMATION

ReIMAGINE Prostate cancer screening is a single site screening study at University College London Hospital NHS Foundation Trust (UCLH). The study will assess the feasibility of MRI as a screening tool for prostate care and determine the prevalence of MRI defined suspicious lesions and cancer in men across a spectrum of PSA results. Investigators from UCLH designed the study and University College London (UCL) is the study sponsor (123874).

The study opened to recruitment on 22nd October 2019, and was paused for five months (March to August 2020) due to the Coronavirus pandemic. Recruitment is expected to compelte in January 2021. Ethical Approval was granted by London – Stanmore Research Ethics Committee Heath Research Authority (reference 19/LO/1129). The study is registered ClinicalTrials.gov identifier NCT04063566.

Study results will be published in peer reviewed journals after completion of data analysis and used to inform the design of a multi-centre screening study in the UK.

This study is supported by the Medical Research Council (MRC) [grant number MR/R014043/1] and Cancer Research UK (CRUK). The CI and PIs have no financial interests in the products or companies involved in the study.

For peer review only

STUDY OBJECTIVES

Primary

The primary objective of the study is to determine the acceptance rate of an invitation for a screening prostate MRI in men who do not have a prostate cancer diagnosis. The study will also seek to determine the prevalence of MRI defined suspicious lesions in men accepting a screening invitation and the subsequent confirmed presence of cancer for those men having biopsy as a result of their MRI findings.

Secondary

Secondary objectives are:

- To determine the proportion of men ineligible due to prior prostate cancer diagnosis.
- To determine the proportion of men who screen negative on MRI and PSA density.
- To determine the proportion of men who screen positive on PSA density alone.
- To determine the proportion of men who screen positive on MRI alone.

STUDY POPULATION

Men aged 50 to 75 years old with no previous prostate cancer diagnosis will be the target population. Study participants will be identified through general practitioner (GP) practices who will act as participant identification centres (PIC's). Potentially eligible men within the London Cancer Networks participating in the study will be sent a personalised invitation letter from their own GP. GP practice staff, supported by clinical research network (CRN) staff, will apply eligibility criteria to their existing patient databases. Database searches will include coding to exclude men who do not meet the entry criteria, or have one or more exclusion criteria.

Invitations will be sent in batches of 50-100, to randomly selected men, until 300 men have had their MRI scan. Respondents will be asked to contact study staff directly and given the opportunity to ask further questions and taken though a checklist for to assess eligibility. The responders who meet all the inclusion criteria and none of the exclusion criteria will be invited to attend a study visit for formal screening, written consent and the study interventions at a single study visit. Table 1 shows inclusion and exclusion criteria and Figure 1 outlines study participant flow.

For peer review only

Inclusion Criteria
**Men aged 50-75 years**
**No prior prostate cancer diagnosis or treatment**
**Willing and able to provide written informed consent**

Exclusion Criteria
**Contraindication for MRI scanning (as assessed by the MRI safety questionnaire of the PET/MRI department) which includes but is not limited to: Intracranial aneurysm clips or other metallic objects; Intra-orbital metal fragments that have not been removed; Pacemakers or other implanted cardiac rhythm management devices and non-MRI compatible heart valves; Inner ear implants; and History of claustrophobia**
**Men who require assisted living e.g. care home living**
**Dementia or other neurological condition meaning participant lacks the capacity to consent**

Table 1: Eligibility Criteria for the ReIMAGINE Prostate Cancer Screening Study.

For peer review only

STUDY DESIGN

Screening Procedures

All men recruited to the ReIMAGINE prostate cancer screening study will receive a prostate specific antigen (PSA) blood test and prostate MRI.

Screening Visit

Following signed consent, baseline data will be collected directly from the participant or their medical/GP records including medical history, demographic information including ethnicity and family history of prostate cancer, social information including marital status and concomitant medications (use of any anti-androgenic medications, in particular 5 alpha-reductase inhibitors in the last 5 years will be specifically asked).

Prostate Specific Antigen (PSA) blood test

This will be done in a standard blood draw by study staff and processed via standard UCLH biochemistry procedures.

MRI Scan

The study MRI will consist of axial T2-weighted, diffusion-weighted and research specific T2 exploratory acquisitions with a total scan time of less than 20 minutes for each participant (see Appendix I for the study MRI protocol). It will be carried out in a 3 Tesla scanner with the participant in the supine position. No endorectal coils will be used. The clinical sequences performed for the bp-MRI study include T2-weighted axial turbo spin echo and diffusion-weighted imaging using a high *b* value of 2000 s/mm^2. The clinical protocol acquisition time will be less than 10 minutes.

Men will undergo exploratory imaging for acquisition of imaging to derive Luminal Water Fraction (LWF) maps. This sequence is designed to detect and grade prostate cancer by assessing the fractional volume of water in the luminal space with cancer having lower luminal water fractions.[10] The total exploratory imaging protocol time with be less than 10 minutes.

For peer review only

Reporting of the MRI Scan

The MRI scan will be reported by two radiologists blinded to clinical details including the PSA. The primary reporter will report on the prostate volume, lesion presence, lesion location and scan quality. The second reporter will report a binary result of ‘screen positive’ or ‘screen negative’ only, but may also include clinical comments (see Reporting Sheet, Figure 2). When there is disagreement between reporters a third independent report will be requested, again reporting on a binary outcome of ‘screen positive’ or ‘screen negative’. A final report (including the third reporter where applicable) will be completed within 14 days from the date of the MRI scan.

The prostate volume (ml) and an overall whole prostate binary result of MRI screen positive or negative will be given to indicate the presence of clinically significant lesion(s). When a lesion is present further data will be reported including; laterality of the lesion (left / right / both), location of the lesion (transition zone, peripheral zone, both, central zone), location of the lesion (base, mid, apex), and additional comments (seminal vesicle involvement, nodal involvement, metastasis).

The T2 exploratory sequence imaging will be analysed retrospectively, qualitatively and quantitatively, for concordance against the clinical bp-MRI.[10] Scans will be reported on the hospital records system (EPIC) and the result extracted when available and entered onto the study database against the participant’s unique participant ID number. Each radiologist should refer to the MRI Standard Operating Procedure (SOP).

Cases where MRI scans could not be done / are not readable will be noted on the MRI electronic case report form (eCRF). If the MRI quality is categorised as non-diagnostic (e.g. due to artefacts from air in the rectum distorting images), screening status will be determined by PSA density alone.

Post-Screening

The MRI reports and PSA result will be reviewed by a trained Urologist no more than 3 weeks after the MRI scan is performed. Men will be deemed ‘screen positive’ if the MRI reports a suspicious visible clinically significant lesion, and/or their PSA density is ≥ 0.12ng/ml. A structured letter of the screening results will be sent to the participant and GP. GPs will be advised that ‘screen positive’ men are referred to secondary care using a suspected cancer pathway referral for an appointment within two weeks in line with National Institute for Health Care and Excellece (NICE) guidelines. Men that ‘screen negative’ will exit the study and no further data will be collected. A letter will be sent to the patient and their GP.

For peer review only

Post Referral Follow Up and Data Collection

Screen positive men will be tracked to ensure follow through with National Health Service (NHS) referral and to gather data from any investigations that may occur as a result of the NHS referral. Further communication with participants and their GP will continue until 3 months after the recommendation letter is sent, to ascertain additional test results.

The number of diagnostic test(s) performed will depend on what is clinically indicated. This could include mpMRI proceeding to a prostate biopsy where clinically indicated. Study staff will also request pseudoanonymised DICOM images of the mpMRI performed at the secondary care NHS trust.

Any adverse events which occur will be recorded in the participant's medical notes and will be reviewed during monitoring visits. The study schedule of events is outlined in Table 2.

**Visit No:**	**Invitation**	**Visit 1**	**Letter of recommendation (non-visit)**	**Healthcare data collection (Non-Visit)**
	Day -1	30 days after acceptance of letter (+/-14 d)	21 days after MRI (+/- 7d)	Up to 3 months after referral to secondary care NHS site (+/- 1 month)
Letter of invitation (up to three attempts)	X			
Informed Consent		X		
Medical History		X		

For peer review only

Baseline data collection		X		
Eligibility confirmation		X		
Registration		X		
PSA		X		
MRI		X		
Result review by Urologist			X	
Structured letter to participant and GP*			X	
Data collection** (NHS diagnostics)				X

An authorised member of the study team will contact the GP of screen positive men, or men themselves, at the below timepoints:

· 1-2 days after the recommendation letter is sent to confirm receipt.

· 2-4 weeks after the recommendation letter is sent to confirm an NHS referral has been actioned.

· 2 or 3 months after the recommendation letter is sent to ensure action was taken at an NHS trust following the GP referral.

*** Diagnostic test(s) will be determined by NHS standard of care following referral to secondary care, and in line with patient agreement / decision. Study staff will request data from the multi parametric MRI (mpMRI) +/- prostate biopsy if clinically indicated.*

For peer review only

Table 2: ReIMAGINE Screening Study Schedule of Assessments. Consent and sample collection (including MRI) will take place on the day of enrolment to the study. Outcomes will be communicated to the participant and their general practitioner before day 28. Healthcare data collection will take place up to 3 months after enrolment.

STATISTICAL CONSIDERATIONS

ReIMAGINE Prostate Cancer Screening is a feasibility study exploring the acceptance rate of MRI screening and the prevalence of MRI lesions and subsequent diagnoses of prostate cancer for men who accept MRI screening. The total number of men invited will be determined by an iterative process of invitation sent in batches until the acceptance rate is determined. From the 300 men who accept the invitation for an MRI scan we anticipate under a third will have suspicious lesions that will prompt referral to NHS secondary care.

There are no formal sample size calculations as this study is being conducted to inform a larger trial that would test the efficacy of MRI based community screening if the feasibility study confirms acceptable levels of uptake. The prevalence of cancer observed for this study would also be used to indicate whether such a screening programme would be worthy of further study in ultimately reducing mortality from prostate cancer. A larger clinical study would be required to test this hypothesis.

For this feasibility study, summary statistics will be used to present the proportions (with 95% confidence intervals) of men who accepted screening and who were found to have suspicious lesions on MRI and the proportion who were subsequently confirmed to have prostate cancer on biopsy. Logistic regression analysis will be used to explore acceptance rates and prevalence of cancer in relation to baseline characteristics.

PATIENT AND PUBLIC INVOLVEMENT (PPI)

The ReIMAGINE project includes a work strand which focuses solely on patient and public involvement (PPI). The work-strand includes a PPI subcommittee which meets every three months. The committee consists of patients, and the wider public, who have been affected by, or have experience of, prostate cancer. The committee meet at least once every three months to inform prioritisation of research outputs, steer media outputs, and assist with development of study materials e.g. participant information videos. All patient facing documents are reviewed by a PPI representative or Chair of the PPI Sub-Committee, and its members.

For peer review only

Patient and public feedback was sought ahead of finalising the screening study design. 17 prostate cancer patients and relatives were invited to join study focus groups where the study protocol was reviewed. Suggestions largely centred around the strategy for communicating screen status to participants and whether this should be done in writing, face to face with study staff or via the participants' General Practitioner (GP). A further focus group was held with GPs to note their feedback regarding study design. The study protocol was refined to reflect the suggestions of both PPI subcommittee and focus group members, as well as GPs.

The PPI team has been responsible for the development of the ReIMAGINE Consortium website (https://www.reimagine-pca.org). The website hosts a range of patient and media information resources relevant to each work strand of the consortium, including the screening study. Resources include details of the consortium directors, consortium partners, and links to participant and public information videos. Additional COVID-19 specific resources have been provided on the website since the study resumed recruitment in August 2020, and outline the steps taken to reduce the risk of COVID-19 exposure during a study visit.

The ReIMAGINE Screening Study Group is outlined in Appendix II.

DISCUSSION AND LIMITATIONS

Few screening and diagnostic paradigms in medicine have remained as stagnant for as long as that of prostate cancer. While large population-based screening studies relying on PSA in the USA, Europe and the UK have showed that they may have the potential to reduce prostate cancer mortality the impact of screening on overall mortality is far less compelling. In addition, prostate cancer screening using PSA and TRUS biopsy is associated with an increased risk of over diagnosis comprising 20-50% of cancers detected.[11] These 'over diagnosed' cancers that would not have presented clinically within the man's lifetime still lead to treatments that can result in significant side effects, particularly on continence and sexual function.

High quality studies have now shown a role for multi-parametric MRI prior to biopsy in men at risk of prostate cancer due to a raised PSA or abnormal digital rectal examination.[6, 7] Based on these data, in April 2018 NHS England defined the best timed prostate cancer diagnostic pathway as using MRI before a biopsy decision in all men fit for active treatment.[12] In 2019, NICE guidelines recommend that

For peer review only

men with a negative MRI can choose to avoid immediate biopsy.[13] Some European and US guidelines now also advocate for MRI prior to biopsy.[14, 15]

While the use of MRI has been recommended in those men under consideration for biopsy, the same guidelines currently recommend against its use as a screening test citing insufficient evidence and concerns that the low specificity of MRI in potentially low-risk patients may result in false-positive findings which could lead to an increase in unnecessary biopsies.[14] Further concerns regarding the use of MRI for screening in the US include the cost and inability to independently study MRI in populations where PSA screening is widespread.[14]

Few studies have evaluated the use of prostate MRI as a population screening test for prostate cancer. As such, to date the majority of data used in support of such an endeavor is extrapolated from MRI use in already at-risk patients with abnormal PSA. A Canadian pilot study, by Nam et al conducted in a group of 47 unselected men from the general population found that mpMRI was better to able to predict prostate cancer than PSA (OR 2.7, 95% CI 1.4-5.4, p = 0.004 vs OR 1.1, 95% CI 0.9-1.4, p = 0.21).[16] This pilot study was used to inform a larger trial using biparametric (bpMRI) that currently ongoing (NCT02799303). In the UK, the Prostate Cancer Screening Trial Using Imaging trial (PROSTAGRAM) utilizing bpMRI (NCT03702439) has completed recruitment and presented initial results, whilst formal publication is awaited.[17] The use of an MRI score > 4 led to a positive test in 10.6% of the 411 screened men, with 11 clinically significant and 5 clinically insignificant cancers found, leading to a sensitivity of 65% and a specificity of 82%. PROSTAGRAM and the Canadian study differ from the current study in that men are excluded from those studies if they have had a recent PSA or previous prostate biopsy. Each study had a recruitment policy that encouraged participation from interested men who had heard about the study, whereas the approach in ReIMAGINE screening mirrors formal UK screening programmes of invitation via GP practice alone.

In addition to reporting on the prevalence of MRI defined suspicious lesions and the subsequent confirmed presence of cancer the present study seeks to determine the acceptance for the use MRI as screening tool by men that are invited to the study. This information will be important to report to determine the feasibility of an MRI based screening program.

There are limitations of the present study. The first potential limitation of this study is that it does not exclude those men with previous PSA testing, history of negative prostate biopsies, or those with previous prostate MRIs. However, in doing so this heterogenous group should more accurately

For peer review only

reflect the general population and improve external validity. There are no exclusions for patients on therapy for benign prostate hyperplasia (BPH) or those with previous BPH procedures should again result in a heterogenous group reflective of the intended screening population. The ReIMAGINE prostate cancer screening study will be conducted at single UK centre with extensive experience publication record with mpMRI. As such, the outcomes may not be easily reproducible in less experienced centres.

Conclusion

ReIMAGINE Screening Study is a single center feasibility study exploring the acceptance rate of MRI screening and the prevalence of MRI lesions and subsequent diagnoses of prostate cancer for men who accept MRI screening. It is an important step in working towards further reducing the harms of prostate cancer screening. If this feasibility study confirms acceptable levels of uptake it will be used to inform a larger study that would test the efficacy of MRI based community screening. ReIMAGINE Prostate Cancer Screening is open to recruitment and is expected to complete recruitment in 2020.

For peer review only

For peer review only

Figure 1. ReIMAGINE Prostate Cancer Screening Study flow. Invitations to participate in the study will be sent to randomly selected, eligible men from collaborating general practice (GP) surgeries. Eligibility of responding men will be confirmed during a telephone consult prior to recruitment. On the day of the study visit, men will complete a study consent form, donate blood for PSA testing and undergo a ReIMAGINE Screening Study MRI of the prostate. Those who are screen negative will exit the study. Screen positive participants will be invited to undergo standard of care evaluation within an NHS prostate cancer clinic.

Figure 2: ReIMAGINE Screening Study MRI Reporting Sheets and electronic Case Report Forms. A standardised MRI reporting sheet will be used by each reporter within the study. A) The primary reporter will report on the prostate volume, lesion presence, lesion location and scan quality. B) The second reporter will report a binary result of 'screen positive' or 'screen negative' only, but may also include clinical comments.

For peer review only

Ethics: ReIMAGINE Prostate Cancer Screening has regulatory approval from the Regional Ethics Committee London – Stanmore (reference 19/LO/1129).

Funding: ReIMAGINE Screening is funded by The Medical Research Council, U.K. (MRC), grant number MR/R014043/1 and Cancer Research U.K. (CRUK).

Conflicts of Interest:

Caroline M Moore receives funding from the Prostate Cancer UK, Movember, the Medical Research Council, Cancer Research UK and the NIHR. She receives fees for HIFU proctoring from SonaCare . She has received speaker fees from Astellas, and Jannsen. She carries out research into photodyanamic therapy supported by Spectracure.

Mark Emberton serves as a consultant/educator/trainer to Sonacare Inc., Exact Imaging, Angiodynamics Inc and Profound Medical. Acknowledgements - Mark Emberton receives research support from the United Kingdom's National Institute of Health Research (NIHR) UCLH/UCL Biomedical Research Centre.

Shonit Punwani receives research support from the United Kingdom's National Institute of Health Research (NIHR) UCLH/UCL Biomedical Research Centre.

Francesco Giganti is funded by the UCL Graduate Research Scholarship and the Brahm PhD scholarship in memory of Chris Adams.

Ton Coolen receives funding from Cancer Research UK and is Director of Saddle Point Science Ltd.

For peer review only

1. CancerResearchUK. *Prostate Cancer Statistics*. Available from: https://www.cancerresearchuk.org/health-professional/cancer-statistics/statistics-by-cancer-type/prostate-cancer#heading-Zero. Accessed November 2020.

2. Andriole GL, Crawford DE, Grubb RL, et al. Mortality results from a randomized prostate-cancer screening trial. *New England Journal of Medicine* 2009;360:1310-1319.

3. Schröder FH, Hugosson J, Roobol M, et al. Screening and prostate cancer mortality: results of the European Randomised Study of Screening for Prostate Cancer (ERSPC) at 13 years of follow-up. *The Lancet 2014*;384:2027-2035.

4. Hugosson J, Roobol MJ, Månsson M, et al. A 16-yr Follow-up of the European Randomized study of Screening for Prostate Cancer. *Eur Urol* 2019;76:43-51.

5. Martin RM, Donovan JL, Turner EL, et al. Effect of a low-intensity PSA-based screening intervention on prostate cancer mortality: the CAP randomized clinical trial. *Jama* 2018;319:883-895.

6. Ahmed HU, El-Shater Bosaily A, Brown LC, et al. Diagnostic accuracy of multi-parametric MRI and TRUS biopsy in prostate cancer (PROMIS): a paired validating confirmatory study. *The Lancet* 2017; 389:815-822.

7. Kasivisvanathan V, Rannikko A S, Borghi M, et al. MRI-targeted or standard biopsy for prostate-cancer diagnosis. *New England Journal of Medicine* 2018;378:1767-1777.

8. Rouvière O, Puech P, Renard-Penna R, et al. Use of prostate systematic and targeted biopsy on the basis of multiparametric MRI in biopsy-naive patients (MRI-FIRST): a prospective, multicentre, paired diagnostic study. *The Lancet Oncology* 2019;20:100-109.

9. van der Leest M, Cornel E, Israël B, et al. Head-to-head comparison of transrectal ultrasound-guided prostate biopsy versus multiparametric prostate resonance imaging with subsequent magnetic resonance-guided biopsy in biopsy-naïve men with elevated prostate-specific antigen: a large prospective multicenter clinical study. *European Urology* 2019;75:570-578.

10. Devine W, Giganti F, Johnston E, et al. Simplified luminal water imaging for the detection of prostate cancer from multiecho T2 MR images. *Journal of Magnetic Resonance Imaging* 2019;50:910-917.

11. Fenton, JJ, Weyrich MS, Durbin S, et al. Prostate-specific antigen–based screening for prostate cancer: evidence report and systematic review for the US Preventive Services Task Force. *Jama* 2018; 319:1914-1931.

For peer review only

12. Implementing a timed prostate cancer diagnostic pathway. NHS Cancer Programme Operations Information. NHS England, 2018. Available from: https://www.england.nhs.uk/wp-content/uploads/2018/04/implementing-timed-prostate-cancer-diagnostic-pathway.pdf. Accessed November 2020.

13. NICE, *Prostate cancer: diagnosis and management NICE guideline [NG131].* 2019. Available from: https://www.nice.org.uk/guidance/ng131. Accessed November 2020.

14. Bjurlin MA, Carroll PR, Eggener S et al. Update of the Standard Operating Procedure on the Use of Multiparametric Magnetic Resonance Imaging for the Diagnosis, Staging and Management of Prostate Cancer. *J Urol.* 2020;203:706-712.

15. Lam TBL, MacLennan S, Willemse PM et al. EAU-EANM-ESTRO-ESUR-SIOG Prostate Cancer Guideline Panel Consensus Statements for Deferred Treatment with Curative Intent for Localised Prostate Cancer from an International Collaborative Study (DETECTIVE Study). *Eur Urol. 2019;* 76:790-813.

16. Nam RK, Wallis CJ, Stojcic-Bendavid J, et al. A Pilot Study to Evaluate the Role of Magnetic Resonance Imaging for Prostate Cancer Screening in the General Population. *J Urol*. 2016;196:361-6.

17. Eldred-Evans D, Burak P, Connor MJ et al. Population-based prostate cancer screening using a prospective, blinded, paired screen-positive comparison of PSA and fast MRI: The IP1-PROSTAGRAM study. *Journal of Clinical Oncology* 2020; 38:5513-5513

For peer review only

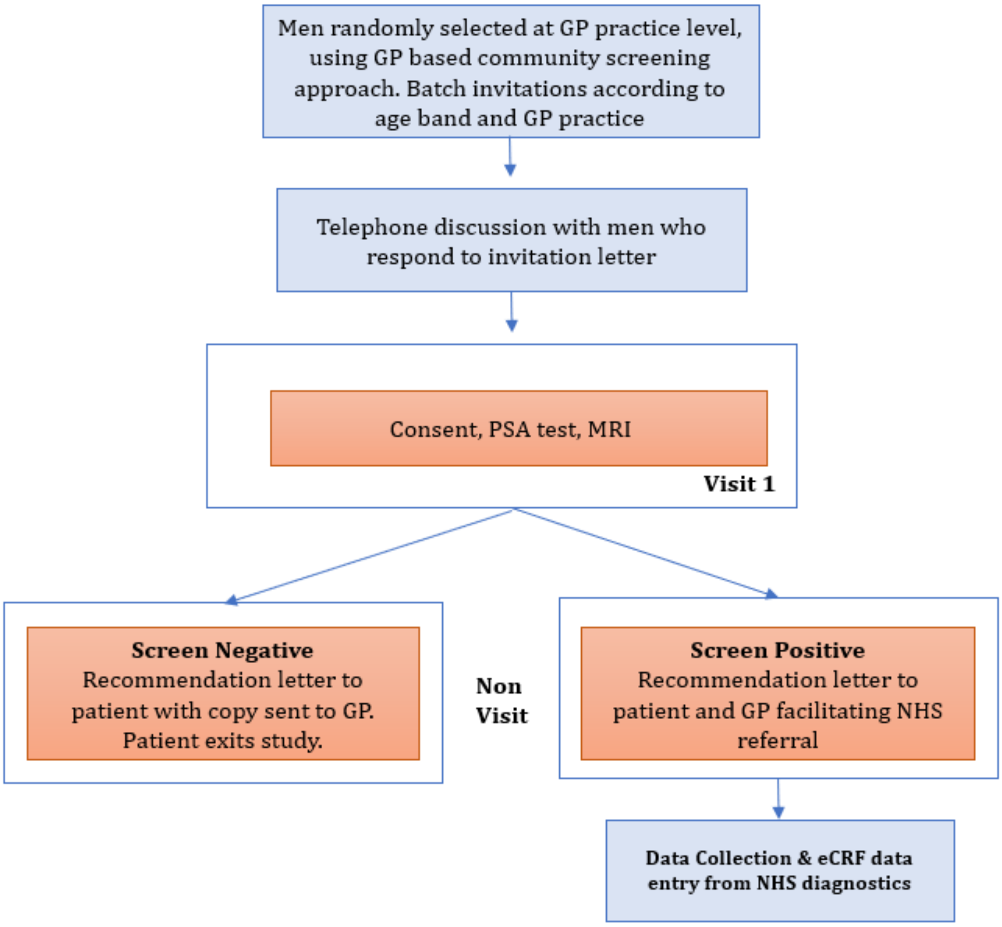

For peer review only - http://bmjopen.bmj.com/site/about/guidelines.xhtml

NHS
University College London Hospitals
NHS Foundation Trust

reimagine
Prostate Cancer Screening

**Patient name**	
**Hospital no.**	
**MRI date**	

**MRI REPORT**	
**REPORTER 1:**	
MRI Prostate volume (ml)	
MRI scan result	☐ Screen Positive ☐ Screen Negative
Lesion laterality	☐ Left ☐ Right ☐ Both
Lesion location 1	☐ TZ ☐ PZ ☐ CZ
Lesion location 2	☐ Base ☐ Mid ☐ Apex
Report comments	
MRI scan quality	☐ Diagnostic ☐ Non - Diagnostic
Date & Time of Report	

N.B. If there is a visible concordant positive lesion on the MRI (seen on both T2 & b2000) please report as positive on a non-diagnostic scan i.e. the whole prostate is not visible due to artefact(s).

Please use the following reporting criteria to ascribe screen positive or screen negative status:

Axial T2 (Likert)	Axial b2000 (Likert)	Screen
focal 4-5/5 or diffuse 3/5	focal 4-5/5	Positive
focal 4-5/5	focal 1-2/5 or diffuse 3/5	Negative
diffuse 3/5	1-2/5 or Likert diffuse 3/5	Negative
1-2/5	ANY	Negative
ANY	1-2/5	Negative
Non-diagnostic (artefact)	1-2/5 or diffuse 3/5	Negative
1-2/5 or diffuse 3/5	Non-diagnostic (artefact)	Negative
4-5/5	Non-diagnostic (artefact)	Non-diagnostic (Recall)
Non-diagnostic (artefact)	4-5/5	Non-diagnostic (Recall)
Non-diagnostic (artefact)	Non-diagnostic (artefact)	Non-diagnostic (No recall)

ReIMAGINE PCa Screening (IRAS 251167)
MRI Reporter 1 CRF
V1.0, dated 1st November 2019

uclh University College Hospital | National Hospital for Neurology and Neurosurgery | Eastman Dental Hospital | Royal National Throat, Nose and Ear Hospital | Royal London Hospital for Integrated Medicine

A

NHS
University College London Hospitals
NHS Foundation Trust

relimagine
Prostate Cancer Screening

**Patient name**	
**Hospital no.**	
**MRI date**	/ /

**REPORTER 2 / 3:**		
MRI scan result	☐ Screen positive	☐ Screen negative
MRI scan quality	☐ Diagnostic	☐ Non-Diagnostic

Comments:

Date & Time of Report:

Please use the following reporting criteria to ascribe screen positive or screen negative status:

Axial T2 (Likert)	Axial b2000 (Likert)	Screen
focal 4-5/5 or diffuse 3/5	focal 4-5/5	Positive
focal 4-5/5	focal 1-2/5 or diffuse 3/5	Negative
diffuse 3/5	1-2/5 or Likert diffuse 3/5	Negative
1-2/5	ANY	Negative
ANY	1-2/5	Negative
Non-diagnostic (artefact)	1-2/5 or diffuse 3/5	Negative
1-2/5 or diffuse 3/5	Non-diagnostic (artefact)	Negative
4-5/5	Non-diagnostic (artefact)	Non-diagnostic (Recall)
Non-diagnostic (artefact)	4-5/5	Non-diagnostic (Recall)
Non-diagnostic (artefact)	Non-diagnostic (artefact)	Non-diagnostic (No recall)

ReIMAGINE PCa Screening (IRAS 251167)
MRI Reporter 2/3 CRF
V1.1, dated 20th Nov 2019

uclh University College Hospital | National Hospital for Neurology and Neurosurgery | Eastman Dental Hospital | Royal National Throat, Nose and Ear Hospital | Royal London Hospital for Integrated Medicine

B

only

Appendix I: MRI protocol

All studies will be performed with the same protocol on a single 3 T scanner (Achieva®, Philips Healthcare, Netherlands) using a 32-channel pelvic-phased array coil and a bi-parametric acquisition protocol. Briefly, sequences will include an axial turbo spin echo and axial diffusion weighted imaging using a high b value at 2000 s mm–2. Exemplar sequence parameters are illustrated in the table below. Total protocol acquisition time <10 minutes.

Clinical bi-parametric MRI at 3T

Parameter	Repetition time (ms)	Echo time (ms)	Flip angle (degree)	Orientation	Slice thickness (mm)	Matrix size	Field of view (mm)	Fat suppression	Time for scan (min)
**T_2 axial**	5407	100	90	Axial	3	300 × 290	180	No	05:13
**DWI b 2000 s mm^{-2}**	2000	78	90	Axial	5	168 × 169	220	SPIR	03:40

The above images will be prospectively reported by radiologists to ascribe a positive/negative screen result as described in the ReIMAGINE Prostate Cancer Screening study protocol. Reports will be generated in UCLH EPIC using a study specific reporting template.

At the same sitting, patients will undergo exploratory imaging for acquisition of imaging to derive Luminal Water Fraction (LWF) maps. Imaging will be performed with the same scanner and coil setup. An 8-echo multi-echo spin echo sequence will be employed to provide axial imaging of the prostate. Exemplar sequence parameters are illustrated in the table below. Total exploratory imaging protocol time < 10 minutes.

For peer review only - http://bmjopen.bmj.com/site/about/guidelines.xhtml

Exploratory LWF MRI at 3T

Parameter	Repetition time (ms)	Echo time/spacing (ms)	Flip angle (degree)	Orientation	Slice thickness (mm)	Matrix size	Field of view (mm)	Fat suppression	Time for scan (min)
**LWF Imaging**	8956	31.25/31.25	90	Axial	4	300 × 290	180	No	05:13

Positive/negative screen status/quantitative analysis and concordance with the bi-parametric MRI will be performed. Reports will not be made available to the clinical team.

For peer review only

Appendix II – The ReIMAGINE Screening Study Group

**Trial Sponsor:**	University College London (UCL)
**Trial Coordination:**	UCL Urology, Research Department of Targeted Intervention, Division of Surgery and Interventional Science
**Funders:**	The Medical Research Council, U.K. (MRC), grant number MR/R014043/1 and Cancer Research U.K. (CRUK)

Trial Management Group

**Caroline M Moore**	Co-Chief Investigator, Urologist - UCLH / UCL
**Mark Emberton**	Co-Chief Investigator, Urologist - UCLH / UCL
**Shonit Punwani**	Co-Investigator, Radiologist - UCLH / UCL
**Neil McCartan**	Clinical Project Management – UCL
**Louise Brown**	Co-Investigator – UCL MRC Clinical Trials Unit
**Mieke Van Hemelrijck**	Co-Investigator, Cancer Epidemiologist - KCL
**Ton Coolen**	Co-Investigator, Applied Mathematics - London Institute for Mathematical Sciences
**Aida Santaollala**	Cancer Epidemiology Group – KCL
**Teresa Marsden**	Clinical Research Fellow, Urology – UCLH / UCL
**Harbir Sidhu**	Consultant Radiologist – UCLH
**Douglas Kopcke**	Honorary Consultant Radiologist - UCLH
**Giorgio Brembilla**	Clinical Research Fellow, Radiology – UCLH / UCL
**Francesco Giganti**	Clinical Research Fellow, Radiology – UCLH / UCL

ReIMAGINE Screening Study Group

**Joanna Hadley**	Clinical Trial Practitioner – UCL
**Fatima Akbar**	Clinical Trial Practitioner - UCL
**Ged Corbett**	Project Manager – UCL TRO
**Manuel Rodriguez-Justo**	Pathologist – UCLH / UCL
**Elizabeth Isaac**	Imaging Trials Unit - UCLH
**Steve Tuck**	ReIMAGINE Consortium Patient Representative
**Saran Green**	Patient and Public Involvement - KCL
**Charlotte Moss**	Data Management - KCL
**Anna J Haire**	Data Management - KCL

For peer review only

**Teresita Beeston**	Imaging Trials Unit - UCLH
**Joey Clement**	Imaging Trials Unit - UCLH
**Fiona Gong**	Centre for Medical Imaging – UCL
**Lucy Casselton**	Centre for Medical Imaging – UCL
**Anna Wingate**	Laboratory Manager – UCL
**Katerina Soteriou**	Imaging Trials Unit - UCLH
**Charlotte Bevan**	Biological Research Committee - ReIMAGINE
**Paul Boutros**	Biological Research Committee - ReIMAGINE
**Andrew Feber**	Biological Research Committee - ReIMAGINE
**Hayley Whitaker**	Biological Research Committee - ReIMAGINE
**Caroline Dive**	Scientific Advisory Board - ReIMAGINE
**Eytan Domany**	Scientific Advisory Board - ReIMAGINE
**Malcolm Mason**	Scientific Advisory Board - ReIMAGINE
**Anwar Padhani**	Scientific Advisory Board - ReIMAGINE
**Eric Aboagye**	Steering Committee - ReIMAGINE
**Richard Kaplan**	Steering Committee - ReIMAGINE
**Chris Parker**	Steering Committee - ReIMAGINE
**Peter Parker**	Steering Committee - ReIMAGINE

ew only

BMJ Open

ReIMAGINE Prostate Cancer Screening Study – A single centre feasibility study inviting men for prostate cancer screening using MRI.

Journal:	*BMJ Open*
Manuscript ID	bmjopen-2020-048144.R1
Article Type:	Protocol
Date Submitted by the Author:	02-Jun-2021
Complete List of Authors:	Marsden, Teresa; University College London, Division of Surgical and Interventional Sciences; University College London Hospitals NHS Foundation Trust, Department of Urology<br>Lomas, Derek J; Mayo Clinic, Department of Urology<br>McCartan, Neil; University College London, Division of Surgical and Interventional Sciences; University College London Hospitals NHS Foundation Trust, Department of Urology<br>Hadley, Joanna; University College London, UCL Division of Surgical & Interventional Sciences; University College London Hospitals NHS Foundation Trust, Department of Urology<br>Tuck, Steve; University College London, ReIMAGINE Consortium Patient Representative<br>Brown, Louise; University College London, MRC Clinical Trials Unit<br>Haire, Anna ; King's College London, School of Cancer and Pharmaceutical Sciences<br>Moss, Charlotte; King's College London, School of Cancer and Pharmaceutical Sciences<br>Green, Saran; King's College London, School of Cancer and Pharmaceutical Sciences<br>Van Hemelrijck, Mieke; King's College London, School of Cancer and Pharmaceutical Sciences<br>Coolen, Ton; London Institute for Mathematical Sciences<br>Santaolalla, Aida ; King's College London, Cancer Epidemiology Group, Division of Cancer Studies<br>Isaac, Elizabeth; University College London, Centre for Medical Imaging<br>Brembilla, Giorgio; University College London, Centre for Medical Imaging<br>Kopcke, Douglas; University College London Hospitals NHS Foundation Trust, Department of Radiology<br>Giganti, Francesco; University College London, UCL Division of Surgical & Interventional Sciences; University College London Hospitals NHS Foundation Trust, Department of Radiology<br>Sidhu, Harbir; University College London, Centre for Medical Imaging<br>Punwani, Shonit; University College London, Centre for Medical Imaging<br>Emberton, Mark; University College London, Division of Surgery and Interventional Sciences; University College London Hospitals NHS Foundation Trust, Department of Urology<br>Moore, Caroline; University College London, Division of Surgery and Interventional Sciences; University College London Hospitals NHS

	Foundation Trust, Department of Urology
**Primary Subject Heading**:	Urology
Secondary Subject Heading:	Surgery, Research methods, Epidemiology
Keywords:	Magnetic resonance imaging < RADIOLOGY & IMAGING, Prostate disease < UROLOGY, Urological tumours < ONCOLOGY, Epidemiology < ONCOLOGY

SCHOLARONE™
Manuscripts

I, the Submitting Author has the right to grant and does grant on behalf of all authors of the Work (as defined in the below author licence), an exclusive licence and/or a non-exclusive licence for contributions from authors who are: i) UK Crown employees; ii) where BMJ has agreed a CC-BY licence shall apply, and/or iii) in accordance with the terms applicable for US Federal Government officers or employees acting as part of their official duties; on a worldwide, perpetual, irrevocable, royalty-free basis to BMJ Publishing Group Ltd ("BMJ") its licensees and where the relevant Journal is co-owned by BMJ to the co-owners of the Journal, to publish the Work in this journal and any other BMJ products and to exploit all rights, as set out in our licence.

The Submitting Author accepts and understands that any supply made under these terms is made by BMJ to the Submitting Author unless you are acting as an employee on behalf of your employer or a postgraduate student of an affiliated institution which is paying any applicable article publishing charge ("APC") for Open Access articles. Where the Submitting Author wishes to make the Work available on an Open Access basis (and intends to pay the relevant APC), the terms of reuse of such Open Access shall be governed by a Creative Commons licence – details of these licences and which Creative Commons licence will apply to this Work are set out in our licence referred to above.

Other than as permitted in any relevant BMJ Author's Self Archiving Policies, I confirm this Work has not been accepted for publication elsewhere, is not being considered for publication elsewhere and does not duplicate material already published. I confirm all authors consent to publication of this Work and authorise the granting of this licence.

For peer review only

The ReIMAGINE Prostate Cancer Screening Study – Protocol for a single centre feasibility study inviting men for prostate cancer screening using MRI.

Teresa Marsden [1,2], Derek J Lomas [3], Neil McCartan [1,2], Joanna Hadley [1,2], Steve Tuck [4], Louise Brown [5], Anna J Haire [6], Charlotte L Moss [6], Saran Green [6], Mieke Van Hemelrijck [6], Ton Coolen [7], Aida Santaolalla [8], Elizabeth Isaac [9], Giorgio Brembilla [9], Douglas Kopcke [10], Francesco Giganti [1,10], Harbir Sidhu [9], Shonit Punwani [9], Mark Emberton* [1,2],Caroline M Moore* [1,2] on behalf of the ReIMAGINE Study Group.

* Joint senior authors

[1] UCL Division of Surgical & Interventional Sciences, University College London, London, UK

[2] Department of Urology, University College London Hospitals NHS Foundation Trust, London, UK

[3] Department of Urology, Mayo Clinic, Rochester, Minnesota, USA

[4] ReIMAGINE Consortium Patient Representative

[5] MRC Clinical Trials Unit, University College London, London, UK

[6] School of Cancer and Pharmaceutical Sciences, Kings College London, London, UK

[7] London Institute for Mathematical Sciences, 35A South Street, Mayfair, London W1K 2XF

[8] Cancer Epidemiology Group, Division of Cancer Studies, King's College London School of Medicine, London, UK

[9] Centre for Medical Imaging, University College London, London, UK

[10] Department of Radiology, University College London Hospitals NHS Foundation Trust, London, UK

Contributors The study concept and design was conceived by CMM, ME, SP, LB, NMC, ST, SG, AJH, CLM and MVH. JH, TM EI, GB, DK, FG, HS and SP will conduct screening and data collection. Analysis will be performed by LB, MVH, TC, AS, SP and CMM. CMM, NMC, DJL and TM prepared the first draft of the manuscript. All authors provided edits and critiqued the manuscript for intellectual content.

For peer review only - http://bmjopen.bmj.com/site/about/guidelines.xhtml

Correspondence to:

Ms Teresa Marsden BMedSci (Hons), BMBS, MRCS (Eng)

UCL Division of Surgical & Interventional Sciences

3rd Floor, Charles Bell House

43-45 Foley Street

London

W1W 7TS

teresa.marsden@ucl.ac.uk

Running title: ReIMAGINE Prostate Cancer Screening

Total word count: 3992 (word limit 4000)

Keywords: Magnetic Resonance Imaging, mpMRI; Prostate Cancer; Screening; Prostate Specific Antigen, PSA.

Abbreviations: Electronic Case Report Form, eCRF; European Union, EU; General Practitioner (GP); multi-parametric magnetic resonance imaging, mp-MRI; magnetic resonance imaging, MRI; material transfer agreement, MTA; multiparametric magnetic resonance imaging, mpMRI; National Health Service, NHS; prostate cancer, PCa; prostate specific antigen, PSA; Research Ethics Committee, REC; Standard Operating Procedure, SOP; Transrectal Ultrasound, TRUS; University College London, UCL.

For peer review only

Abstract

Introduction: The primary objective of the ReIMAGINE prostate cancer screening study is to explore the uptake of an invitation to prostate cancer screening using magnetic resonance imaging (MRI).

Methods and Analysis: The ReIMAGINE prostate cancer screening study is a prospective single center feasibility study (registration number NCT04063566). Eligible men aged 50 to 75 years with no prior prostate cancer diagnosis or treatment will be identified through general practitioner practices and randomly selected for invitation. Those invited will be offered an MRI scan and prostate specific antigen (PSA) blood test. The screening MRI scan consists of T2-weighted, diffusion-weighted and research specific sequences, without the use of intravenous contrast agents. Men who screen positive on either MRI or PSA density will be recommended to have standard of care (National Health Service) tests for prostate cancer assessment, which includes multi-paremetric MRI. The study will assess the acceptability of an MRI based prostate screening assessment and the prevalence of cancer detected in MRI screened men. Summary statistics will be used to explore baseline characteristics in relation to acceptance rates and prevalence of cancer.

Ethics and Dissemination: ReIMAGINE prostate cancer screening is a single site screening study to assess the feasibility of MRI as a screening tool for prostate cancer. Ethical Approval was granted by London – Stanmore Research Ethics Committee Heath Research Authority (reference 19/LO/1129). Study results will be published in peer reviewed journals after completion of data analysis and used to inform the design of a multi-centre screening study in the UK.

STRENGTHS AND LIMITATIONS OF THE STUDY

- Few studies have evaluated the use of MRI as a population screening test for prostate cancer.
- Recruitment to ReIMAGINE Screening mirrors formal UK screening programmes of invitation via GP practice alone.
- The study will assess the acceptability of MRI as a screening tool, the prevalence of MRI defined suspicious lesions and any subsequent prostate cancer detected as a result of their screening invitation.
- The study does not exclude men with a history of PSA testing, previous negative prostate biopsies or prostate MRIs and therefore provides an heterogenous population representative of the general population.

For peer review only

- Participation identification centres (GP practices) are limited to the London area limiting generalizability of findings.

INTRODUCTION

Prostate cancer is the most common cancer in men and second most common cause of male cancer-related death in the UK.[1] Cancer screening seeks to detect disease before it becomes clinically apparent to allow effective treatment. The UK has no prostate cancer screening programme, but men can ask for a prostate specific antigen (PSA) test via their general practitioner (GP), and men with a raised PSA or an abnormal digital rectal examination (DRE) are encouraged to undergo hospital-based testing.

Several large randomized population-based studies have been conducted to determine the long-term effect of a prostate cancer screening programme on overall and prostate cancer specific mortality. The Prostate, Lung, Colorectal and Ovarian (PLCO) screening study randomized over 76,000 men to annual PSA screening or usual care.[2] It showed no difference in prostate cancer mortality in the screened and control arms but has been criticized because half of the control arm had a PSA test during the study. The European Randomised Screening for Prostate Cancer (ERSPC) study provides 16-year follow up data for 162,389 men (age 55–69), radomised to PSA screening and control groups.[3, 4] Prostate cancer mortality was lower in the screening arm and relative to 13 year data, both the number needed to screen and diagnose fell suggesting a benefit to screening in the longer term. However, the number needed to diagnose remains high at 18, suggesting considerable overdiagnosis. In the UK Cluster randomised trial of PSA testing for prostate cancer (CAP) of 419,582 men, using a single PSA test, followed by a standard transrectal biopsy if the PSA was between 3 and 19.9 ng/ml, there was no difference in prostate cancer mortality in the PSA screened and control arms.[5]

While PSA screening may reduce prostate cancer mortality, it is associated with an increased risk of over-diagnosis. PSA screening using varying protocols fails to discriminate clinically important from unimportant disease, and a significant proportion of PSA-screen detected cancer (at least 45% in large population-based PSA screening studies) is considered low grade. [2-5] As such this remains an undesirable screening approach, due to overdetection and subsequent overtreatment, or unnecessary surveillance and anxiety.

Magnetic Resonance Imaging (MRI) performs better than standard biopsy in men with a clinical suspicion of prostate cancer based on a raised PSA or abnormal DRE. The UK PROMIS study compared

For peer review only - http://bmjopen.bmj.com/site/about/guidelines.xhtml

multiparametric MRI (mpMRI) and 12-core TRUS biopsy with 5mm transperineal mapping biopsy as a gold-standard comparator. Almost twice as many clinically significant cancers were detected on mpMRI as on systematic sampling.[6] The international PRECISION study randomised men to either standard TRUS biopsy or an mpMRI with targeted biopsy alone for those men with a lesion scoring ≥3 on mpMRI.[7] The MRI-based strategy resulted in increased diagnosis of clinically significant prostate cancer (38% vs 26%), with a reduction in detection of indolent disease (9% vs 22%) with 28% of men avoiding biopsy in the MRI arm. Klotz et al have similarly described MR imaging with targeted biopsy as noninferior to systematic biopsy in the detection of clinically important (≥ Gleason Grade Group 2) cancers, whilst avoiding biopsy in more than one-third of men and reducing the diagnosis of clinically insignificant cancer. [8] The MRI First and 4M studies have also confirmed that mpMRI and targeted biopsy do not reduce the detection of clinically significant prostate cancer, but could allow up to 50% of men to avoid biopsy, and significantly reduce over-detection.[9, 10]

The next challenge is to see if the benefits of MRI in men with a suspicion of prostate cancer can be translated to the screening setting.

For peer review only

STUDY INFORMATION

ReIMAGINE Prostate cancer screening is a single site screening study at University College London Hospital NHS Foundation Trust (UCLH). The study will assess the feasibility of MRI as a screening tool for prostate care and determine the prevalence of MRI defined suspicious lesions and cancer in men across a spectrum of PSA results. Investigators from UCLH designed the study and University College London (UCL) is the study sponsor (122665).

The study opened to recruitment on 22nd October 2019, and was paused for five months (March to August 2020) due to the Coronavirus pandemic. Recruitment completed in December 2020. There were two non-substantial amendments to the study during recruitment, each described in detail within Appendix I. This manuscript represents protocol version 2.0 14.05.2020.

Ethical Approval was granted by London – Stanmore Research Ethics Committee Heath Research Authority (reference 19/LO/1129). The study is registered ClinicalTrials.gov identifier NCT04063566. Study results will be published in peer reviewed journals after completion of data analysis and used to inform the design of a multi-centre screening study in the UK.

This study is supported by the Medical Research Council (MRC) [grant number MR/R014043/1] and Cancer Research UK (CRUK). The CI and PIs have no financial interests in the products or companies involved in the study.

For peer review only

STUDY OBJECTIVES

Primary

The primary objective of the study is to determine the acceptance rate of an invitation for a screening prostate MRI in men who do not have a prostate cancer diagnosis. The study will also seek to determine the prevalence of MRI defined suspicious lesions in men accepting a screening invitation and the subsequent confirmed presence of cancer for those men having biopsy as a result of their MRI findings.

Secondary

Secondary objectives are:

- To determine the proportion of men ineligible due to prior prostate cancer diagnosis.
- To determine the proportion of men who screen negative on MRI and PSA density.
- To determine the proportion of men who screen positive on PSA density alone.
- To determine the proportion of men who screen positive on MRI alone.

STUDY POPULATION

Men aged 50 to 75 years old with no previous prostate cancer diagnosis comprise the target population. Full inclusion and exclusion criteria are described in Table 1.

For peer review only

**Inclusion Criteria**
**Men aged 50-75 years**
**No prior prostate cancer diagnosis or treatment**
**Willing and able to provide written informed consent**

**Exclusion Criteria**
**Contraindication for MRI scanning (as assessed by the MRI safety questionnaire of the PET/MRI department) which includes but is not limited to: Intracranial aneurysm clips or other metallic objects; Intra-orbital metal fragments that have not been removed; Pacemakers or other implanted cardiac rhythm management devices and non-MRI compatible heart valves; Inner ear implants; and history of claustrophobia**
**Men who require assisted living e.g. care home living**
**Dementia or other neurological condition meaning participant lacks the capacity to consent**

Table 1: Eligibility Criteria for the ReIMAGINE Prostate Cancer Screening Study.

For peer review only

STUDY DESIGN

Screening and invitation to participate

Potential participants were identified through searchable databases at partner GP surgeries acting as participant identification centres (PICs). There are eight PIC sites within the study, five in North London, two in South London, and one in Ilford, Essex. Three further South London PIC sites had appropriate permissions to participate but this was affected by the COVID-19 pandemic. Our aim was to have a representative population of London GP practices with diversity in both affluence and ethnicity. We partnered with cancer research networks (CRN's) in both North and South London to help facilitate this.

Screening for eligibility followed a three-step process:

Step 1

Each of the participating GP surgeries, using the Egton Medical Information Systems (EMIS), applied a code to exclude men from the search if they were outside the age range of 50-75 or had a previous diagnosis of prostate cancer. This then produced a random list of 120 men that was checked visually by the GP to exclude any addresses related to care home / assisted living facilities. After visual exclusions, the first remaining 100 men were sent a study invitation.

Invitation letters were sent using a docmail® account (http://www.docmail.co.uk/). The template letter of invitation is shown in Figure 1. The invitation letter provides contact details for study staff and requests that interested men contact the study team by telephone or email to complete formal eligibility screening (“*Step 2*”).

Step 2

Responders directly contact the study site. At this timepoint the study team pre-screen the potential participant using an “Eligibility & MRI safety checklist” (Figure 2). This checklist is used to pre-screen for exclusion criteria not identified by the EMIS search, ensure it is safe to proceed with booking of the MRI scan and to confirm the responder is truly registered at one of the participating GP practices (confirmation was sought from the GP practice itself). From August 2020 onwards this checklist was modified to include questions in relation to COVID-19 symptoms and testing.

For peer review only

Step 3

On the day of the study visit, in line with NHS standard practice, a further MRI safety checklist is repeated by an NHS trust employed radiographer. Standard research screening and baseline data collection is performed in parallel to this NHS safety check.

Every recruited man was offered both a study MRI scan and PSA level. Table 1 shows study inclusion and exclusion criteria and Figure 3 outlines study participant flow.

Screening Procedures

All men recruited to the ReIMAGINE prostate cancer screening study will receive a prostate specific antigen (PSA) blood test and prostate MRI.

Screening Visit

Following signed consent, baseline data is collected directly from the participant or their medical/GP records including medical history, demographic information including ethnicity and family history of prostate cancer, social information including marital status and concomitant medications (use of any anti-androgenic medications, in particular 5 alpha-reductase inhibitors in the last 5 years will be specifically asked).

Prostate Specific Antigen (PSA) blood test

This is done in a standard blood draw by study staff and processed via standard UCLH biochemistry procedures.

MRI Scan

The study MRI consists of axial T2-weighted, diffusion-weighted and research specific T2 exploratory acquisitions with a total scan time of less than 20 minutes for each participant (see Appendix II for the study MRI protocol). It is carried out in a 3 Tesla scanner with the participant in the supine position. No endorectal coils are used. The clinical sequences performed for the bp-MRI study include T2-weighted axial turbo spin echo and diffusion-weighted imaging using a high *b* value of 2000 s/mm^2. The clinical protocol acquisition time is less than 10 minutes.

For peer review only

Men will undergo exploratory imaging for acquisition of imaging to derive Luminal Water Fraction (LWF) maps. This sequence is designed to detect and grade prostate cancer by assessing the fractional volume of water in the luminal space with cancer having lower luminal water fractions.[11] The total exploratory imaging protocol time with be less than 10 minutes.

For peer review only

Reporting of the MRI Scan

The MRI scan will be reported by two radiologists blinded to clinical details including the PSA. The primary reporter will report on the prostate volume, lesion presence, lesion location and scan quality. All visible MR lesions will be described on an electronic case report form (eCRF). The second reporter will report a binary result of 'screen positive' or 'screen negative' only, but may also include clinical comments including further description of the lesions present (see Reporting Sheet, Figure 4). When there is disagreement between reporters a third independent report will be requested, again reporting on a binary outcome of 'screen positive' or 'screen negative'. A final report (including the third reporter where applicable) will be completed within 14 days from the date of the MRI scan.

The prostate volume (ml) and an overall whole prostate binary result of MRI screen positive or negative will be given to indicate the presence of clinically significant lesion(s). When a lesion is present further data will be reported including: laterality of the lesion (left / right / both), location of the lesion (transition zone, peripheral zone, both, central zone), location of the lesion (base, mid, apex), and additional comments (seminal vesicle involvement, nodal involvement, metastasis). If more than one lesion is present, the smaller or lower scoring lesions will also be described in the free-text area of the eCRF.

The T2 exploratory sequence imaging will be analysed retrospectively, qualitatively and quantitatively, for concordance against the clinical bp-MRI.[11] Scans will be reported on the hospital records system (EPIC) and the result extracted when available and entered onto the study database against the participant's unique participant ID number. Each radiologist should refer to the MRI Standard Operating Procedure (SOP).

Cases where MRI scans could not be done / are not readable will be noted on the MRI eCRF. If the MRI quality is categorised as non-diagnostic (e.g. due to artefacts from air in the rectum distorting images), this will be noted on the MR reporting eCRF and the screening status determined by PSA density alone.

Post-Screening

The MRI reports and PSA result will be reviewed by a trained Urologist no more than 3 weeks after the MRI scan is performed. Men will be deemed 'screen positive' if the MRI reports a suspicious visible clinically significant lesion, and/or their PSA density is ≥ 0.12ng/ml. A structured letter of the screening results will be sent to the participant and GP. GPs will be advised that 'screen positive' men should be referred to secondary care using a suspected cancer pathway referral for an appointment

For peer review only

within two weeks in line with National Institute for Health Care and Excellece (NICE) guidelines. Men that 'screen negative' will exit the study and no further data will be collected. A letter will be sent to the patient and their GP.

Post Referral Follow Up and Data Collection

Screen positive men will be tracked to ensure follow through with National Health Service (NHS) referral and to gather data from any investigations that may occur as a result of the NHS referral. Further communication with participants and their GP will continue until 3 months after the recommendation letter is sent, to ascertain additional test results.

The number of diagnostic test(s) performed will depend on what is clinically indicated. This could include a standard NHS mpMRI proceeding to a prostate biopsy where clinically indicated. Study staff will also request pseudoanonymised DICOM images of the mpMRI performed at the secondary care NHS trust.

Any adverse events which occur will be recorded in the participant's medical notes and will be reviewed during monitoring visits. The study schedule of events is outlined in Table 2.

**Visit No:**	**Invitation**	**Visit 1**	**Letter of recommendation (non-visit)**	**Healthcare data collection (Non-Visit)**
	Day -1	30 days after acceptance of letter (+/-14 d)	21 days after MRI (+/- 7d)	Up to 3 months after referral to secondary care NHS site (+/- 1 month)
Letter of invitation (up to three attempts)	X			
Informed Consent		X		
Medical History		X		

For peer review only

Baseline data collection		X		
Eligibility confirmation		X		
Registration		X		
PSA		X		
MRI		X		
Result review by Urologist			X	
Structured letter to participant and GP*			X	
Data collection** (NHS diagnostics)				X

**An authorised member of the study team will contact the GP of screen positive men, or men themselves, at the below timepoints:*

· 1-2 days after the recommendation letter is sent to confirm receipt.

· 2-4 weeks after the recommendation letter is sent to confirm an NHS referral has been actioned.

· 2 or 3 months after the recommendation letter is sent to ensure action was taken at an NHS trust following the GP referral.

*** Diagnostic test(s) will be determined by NHS standard of care following referral to secondary care, and in line with patient agreement / decision. Study staff will request data from the multi parametric MRI (mpMRI) +/- prostate biopsy if clinically indicated.*

For peer review only

Table 2: ReIMAGINE Screening Study Schedule of Assessments. Consent and sample collection (including MRI) will take place on the day of enrolment to the study. Outcomes will be communicated to the participant and their general practitioner before day 28. Healthcare data collection will take place up to 3 months after enrolment.

Study amendments

There were two non-substantial amendments over the duration of the study and each is described in detail in Appendix I.

For peer review only

STATISTICAL CONSIDERATIONS

ReIMAGINE Prostate Cancer Screening is a feasibility study exploring the acceptance rate of MRI screening and the prevalence of MRI lesions and subsequent diagnoses of prostate cancer for men who accept MRI screening. The total number of men invited will be determined by an iterative process of invitation sent in batches until the acceptance rate is determined. From the 300 men who accept the invitation for an MRI scan we anticipate that less than one third will have suspicious lesions that will prompt referral to NHS secondary care. This figure correlates with an 18% MRI positivity rate (MRI score 3 – 5 on non-contrast prostate MR) observed within the UK PROSTAGRAM (NCT03702439) screening study. [12]

For this feasibility study, summary statistics will be used to present the proportions (with 95% confidence intervals) of men who accepted screening and who were found to have suspicious lesions on MRI and the proportion who were subsequently confirmed to have prostate cancer on biopsy. Aggregate data (partial postcode data to derive deprivation index scoring, age and ethnicity) will be derived for both participants who consented to the study and all men sent invitations (i.e. inclusive of non-responders) via partner GP practices. Logistic regression analysis will be used to explore acceptance rates and prevalence of cancer in relation to baseline characteristics.

Sample size calculation

There are no formal sample size calculations as this study is being conducted to inform a larger trial that would test the efficacy of MRI based community screening if the feasibility study confirms acceptable levels of uptake. In addition to assessing the acceptance rate of a screening invitation, this feasibility study will aim to determine the prevalence of MRI defined suspicious lesions within the cohort of screening MRIs performed. Work by Nam et al evaluating the feasibility of multiparametric MRI prostate as a as a screening test for prostate cancer, irrespective of PSA level, detected MRI score 4 or 5 lesions in 17 of 47 (36%) men. [13] If we assume half this prevalence rate (15%) in men who consent to ReIMAGINE Screening, as they are selected randomly via GP practices, rather than via a newspaper advert, we will require 300 MRIs in order to identify 45 men with MRI score 4 or 5 lesions.

Ultimately, we plan to use the data from the ReIMAGINE Screening study to inform the design of a randomised controlled trial. Acceptance rates and disease pick-up are both important factors to consider and previous work from Nam et al, and the recently published UK PROSTAGRAM study (NCT03702439), which evaluates biparametric prostate MRI as a screening tool for prostate cancer, will

For peer review only - http://bmjopen.bmj.com/site/about/guidelines.xhtml

inform this further. [12] ReIMAGINE Screening represents early work in this field and its budget is limited, which also constrained our sample size to 300.

Future work

Once available, the results of this feasibility study will be taken into consideration alongside the evidence on which other screening programmes have based a decision to screen. For example, uptake rates of national UK breast, cervical and bowel cancer screening programmes are 74%, 70-73% and 50-58%, respectively. [14] Detection of cancer during UK breast and cervical cancer screening is <1% and 6-7%, respectively. 12-15% of men and 8% of women undergoing a colonoscopy or other investigation following an abnormal bowel cancer screening result in England and Scotland receive a cancer diagnosis. [14] These metrics will act as comparators to the outcome measures of our feasibility study and will be used to inform the design (primary outcome, sample size, duration of follow-up) of any future randomised control trial for MR based prostate cancer screening.

For peer review only

PATIENT AND PUBLIC INVOLVEMENT (PPI)

The ReIMAGINE project includes a work strand which focuses solely on patient and public involvement (PPI). The work-strand includes a PPI subcommittee which meets every three months. The committee consists of patients, and the wider public, who have been affected by, or have experience of, prostate cancer. The committee meet at least once every three months to inform prioritisation of research outputs, steer media outputs, and assist with development of study materials e.g. participant information videos. All patient facing documents are reviewed by a PPI representative or Chair of the PPI Sub-Committee, and its members.

Patient and public feedback was sought ahead of finalising the screening study design. 17 prostate cancer patients and relatives were invited to join study focus groups where the study protocol was reviewed. Suggestions largely centred around the strategy for communicating screen status to participants and whether this should be done in writing, face to face with study staff or via the participants' General Practitioner (GP). A further focus group was held with GPs to note their feedback regarding study design. The study protocol was refined to reflect the suggestions of both PPI subcommittee and focus group members, as well as GPs.

The PPI team has been responsible for the development of the ReIMAGINE Consortium website (https://www.reimagine-pca.org). The website hosts a range of patient and media information resources relevant to each work strand of the consortium, including the screening study. Resources include details of the consortium directors, consortium partners, and links to participant and public information videos. Additional COVID-19 specific resources have been provided on the website since the study resumed recruitment in August 2020, and outline the steps taken to reduce the risk of COVID-19 exposure during a study visit.

The ReIMAGINE Screening Study Group is outlined in Appendix III.

For peer review only

DISCUSSION AND LIMITATIONS

Few screening and diagnostic paradigms in medicine have remained as stagnant for as long as that of prostate cancer. While large population-based screening studies relying on PSA in the USA, Europe and the UK have showed that they may have the potential to reduce prostate cancer mortality the impact of screening on overall mortality is far less compelling. In addition, prostate cancer screening using PSA and TRUS biopsy is associated with an increased risk of over diagnosis comprising 20-50% of cancers detected.[15] These 'over diagnosed' cancers that would not have presented clinically within the man's lifetime still lead to treatments that can result in significant side effects, particularly on continence and sexual function.

High quality studies have now shown a role for multi-parametric MRI prior to biopsy in men at risk of prostate cancer due to a raised PSA or abnormal digital rectal examination.[6, 7] Based on these data, in April 2018 NHS England defined the best timed prostate cancer diagnostic pathway as using MRI before a biopsy decision in all men fit for radical treatment.[16] In 2019, NICE guidelines recommended that men with a negative MRI can choose to avoid immediate biopsy.[17] Some European and US guidelines now also advocate for MRI prior to biopsy.[18, 19]

While the use of MRI has been recommended in those men under consideration for biopsy, the same guidelines currently recommend against its use as a screening test citing insufficient evidence and concerns that the low specificity of MRI in potentially low-risk patients may result in false-positive findings which could lead to an increase in unnecessary biopsies.[18] Further concerns regarding the use of MRI for screening in the US include the cost and inability to independently study MRI in populations where PSA screening is widespread.[18]

Few studies have evaluated the use of prostate MRI as a population screening test for prostate cancer. As such, to date the majority of data used in support of such an endeavor is extrapolated from MRI use in already at-risk patients with abnormal PSA. A Canadian pilot study, by Nam et al conducted in a group of 47 men from the general population, recruited via newspaper advert, found that mpMRI was better to able to predict prostate cancer than PSA (OR 2.7, 95% CI 1.4-5.4, p = 0.004 vs OR 1.1, 95% CI 0.9-1.4, p = 0.21). [13] This pilot study was used to inform a larger trial using biparametric (bpMRI) that currently ongoing (NCT02799303). In the UK, the Prostate Cancer Screening Trial Using Imaging trial (PROSTAGRAM) utilising bpMRI (NCT03702439) found that the use of an MRI score > 4 on screening bpMRI led to a positive test in 10.6% of the 411 screened men, with 11 clinically significant and 5 clinically insignificant cancers found, leading to a sensitivity of 65% and a specificity of 82%. [12] PROSTAGRAM and the Canadian study differ from the current study in that men are excluded from

For peer review only

those studies if they have had a recent PSA or previous prostate biopsy. Each study had a recruitment policy that encouraged participation from interested men who had heard about the study, whereas the approach in ReIMAGINE screening mirrors formal UK screening programmes of invitation via GP practice alone.

In addition to reporting on the prevalence of MRI defined suspicious lesions and the subsequent confirmed presence of cancer the present study seeks to determine the acceptance for the use MRI as screening tool by men that are invited to the study. This information will be important to report to determine the feasibility of an MRI based screening program.

There are limitations of the present study. The first potential limitation of this study is that it does not exclude those men with previous PSA testing, history of negative prostate biopsies, or those with previous prostate MRIs. However, in doing so this heterogenous group should more accurately reflect the general population and improve external validity. Second, there are no exclusions for patients on therapy for benign prostate hyperplasia (BPH) or those with previous BPH procedures should again result in a heterogenous group reflective of the intended screening population. Third, the study provides limited information on the feasibility of a randomised controlled trial (RCT). For example, an invitation letter which results in a good response during a feasibility study may not generate such a good response for an RCT during which a proportion of respondents would be allocated to no screening. A further consideration is that the ReIMAGINE prostate cancer screening study will be conducted at single UK centre with extensive clinical experience of mpMRI prostate. As such, the outcomes may not be easily reproducible in less experienced centres.

Conclusion

ReIMAGINE Screening Study is a single center feasibility study exploring the acceptance rate of MRI screening and the prevalence of MRI lesions and subsequent diagnoses of prostate cancer for men who accept MRI screening. It is an important step in working towards further reducing the harms of prostate cancer screening. If this feasibility study confirms acceptable levels of uptake it will be used to inform a larger study that would test the efficacy of MRI based community screening. ReIMAGINE Prostate Cancer Screening completed recruitment in December 2020.

For peer review only

Figure 1: ReIMAGINE Screening Study Invitation to Participate. Potential participants will be identified through searchable databases at partner GP surgeries participating in the study and acting as participant identification centres (PICs). Invitation letters will be sent out by each PIC using a docmail® account (http://www.docmail.co.uk/) in batches of 50 - 100, to randomly selected eligible men, until 300 men have had their MRI scan. The invitation letter provides contact details for study staff. Interested men are asked to contact the team to complete formal eligibility screening and enrol to the study. The template letter of invitation distributed by PICs is included in this figure.

Figure 2: ReIMAGINE Screening study Eligibility and MRI Safety checklist. Following an invitation letter, interested men are invited to contact the ReIMAGINE Screening study team either by telephone or email to complete formal eligibility screening according to the ReIMAGINE Screening Study Eligibility and MRI Safety Checklist. This checklist is used to pre-screen for exclusion criteria not identified by initial searches at partner GP practices, to ensure it is safe to proceed with booking of the MRI scan and to confirm the responder is truly registered at one of the participating GP practices (confirmation is provided by the GP practice itself). From August 2020 onwards this checklist was modified to include questions in relation to COVID-19 symptoms and testing.

Figure 3: ReIMAGINE Prostate Cancer Screening Study flow. Invitations to participate in the study will be sent to randomly selected, eligible men from collaborating general practice (GP) surgeries. Eligibility of responding men will be confirmed during a telephone consult prior to recruitment. On the day of the study visit, men will complete a study consent form, donate blood for PSA testing and undergo a ReIMAGINE Screening Study MRI of the prostate. Those who are screen negative will exit the study. Screen positive participants will be invited to undergo standard of care evaluation within an NHS prostate cancer clinic.

Figure 4: **ReIMAGINE Screening Study MRI Reporting Sheets and electronic Case Report Forms.** A standardised MRI reporting sheet will be used by each reporter within the study. The primary reporter will report on the prostate volume, lesion presence, lesion location and scan quality. The second reporter will report a binary result of ‘screen positive’ or ‘screen negative’ only, but may also include clinical comments.

For peer review only

Ethics: ReIMAGINE Prostate Cancer Screening has regulatory approval from the Regional Ethics Committee London – Stanmore (reference 19/LO/1129).

Funding: ReIMAGINE Screening is funded by The Medical Research Council, U.K. (MRC), grant number MR/R014043/1 and Cancer Research U.K. (CRUK).

Conflicts of Interest:

Caroline M Moore receives funding from the Prostate Cancer UK, Movember, the Medical Research Council, Cancer Research UK and the NIHR. She receives fees for HIFU proctoring from SonaCare . She has received speaker fees from Astellas, and Jannsen. She carries out research into photodyanamic therapy supported by Spectracure.

Mark Emberton serves as a consultant/educator/trainer to Sonacare Inc., Exact Imaging, Angiodynamics Inc and Profound Medical. Acknowledgements - Mark Emberton receives research support from the United Kingdom's National Institute of Health Research (NIHR) UCLH/UCL Biomedical Research Centre.

Shonit Punwani receives research support from the United Kingdom's National Institute of Health Research (NIHR) UCLH/UCL Biomedical Research Centre.

Francesco Giganti is funded by the UCL Graduate Research Scholarship and the Brahm PhD scholarship in memory of Chris Adams.

Ton Coolen receives funding from Cancer Research UK and is Director of Saddle Point Science Ltd.

For peer review only

1. CancerResearchUK. *Prostate Cancer Statistics*. Available from: https://www.cancerresearchuk.org/health-professional/cancer-statistics/statistics-by-cancer-type/prostate-cancer#heading-Zero. Accessed November 2020.

2. Andriole GL, Crawford DE, Grubb RL, et al. Mortality results from a randomized prostate-cancer screening trial. *New England Journal of Medicine* 2009;360:1310-1319.

3. Schröder FH, Hugosson J, Roobol M, et al. Screening and prostate cancer mortality: results of the European Randomised Study of Screening for Prostate Cancer (ERSPC) at 13 years of follow-up. *The Lancet 2014*;384:2027-2035.

4. Hugosson J, Roobol MJ, Månsson M, et al. A 16-yr Follow-up of the European Randomized study of Screening for Prostate Cancer. *Eur Urol* 2019;76:43-51.

5. Martin RM, Donovan JL, Turner EL, et al. Effect of a low-intensity PSA-based screening intervention on prostate cancer mortality: the CAP randomized clinical trial. *Jama* 2018;319:883-895.

6. Ahmed HU, El-Shater Bosaily A, Brown LC, et al. Diagnostic accuracy of multi-parametric MRI and TRUS biopsy in prostate cancer (PROMIS): a paired validating confirmatory study. *The Lancet* 2017; 389:815-822.

7. Kasivisvanathan V, Rannikko A S, Borghi M, et al. MRI-targeted or standard biopsy for prostate-cancer diagnosis. *New England Journal of Medicine* 2018;378:1767-1777.

8. Klotz L, Chin J, Black PC, et al. Comparison of Multiparametric Magnetic Resonance Imaging-Targeted Biopsy With Systematic Transrectal Ultrasonography Biopsy for Biopsy-Naive Men at Risk for Prostate Cancer: A Phase 3 Randomized Clinical Trial. *JAMA Oncology.* 2021;7:534-42.

9. Rouvière O, Puech P, Renard-Penna R, et al. Use of prostate systematic and targeted biopsy on the basis of multiparametric MRI in biopsy-naive patients (MRI-FIRST): a prospective, multicentre, paired diagnostic study. *The Lancet Oncology* 2019;20:100-109.

10. van der Leest M, Cornel E, Israël B, et al. Head-to-head comparison of transrectal ultrasound-guided prostate biopsy versus multiparametric prostate resonance imaging with subsequent magnetic resonance-guided biopsy in biopsy-naïve men with elevated prostate-specific antigen: a large prospective multicenter clinical study. *European Urology* 2019;75:570-578.

11. Devine W, Giganti F, Johnston E, et al. Simplified luminal water imaging for the detection of prostate cancer from multiecho T2 MR images. *Journal of Magnetic Resonance Imaging* 2019;50:910-917.

For peer review only

12. Eldred-Evans D, Burak P, Connor MJ, et al. Population-Based Prostate Cancer Screening With Magnetic Resonance Imaging or Ultrasonography: The IP1-PROSTAGRAM Study. *JAMA Oncol*ogy. 2021;7:395-402.

13. Nam RK, Wallis CJ, Stojcic-Bendavid J, et al. A Pilot Study to Evaluate the Role of Magnetic Resonance Imaging for Prostate Cancer Screening in the General Population. *J Urol*. 2016;196:361-6.

14. UK Cancer Research: *Cancer screening and diagnosis statistics*. https://www.cancerresearchuk.org/health-professional/cancer-screening-and-diagnosis-statistics. Accessed May 2021.

15. Fenton, JJ, Weyrich MS, Durbin S, et al. Prostate-specific antigen–based screening for prostate cancer: evidence report and systematic review for the US Preventive Services Task Force. *Jama* 2018; 319:1914-1931.

16. Implementing a timed prostate cancer diagnostic pathway. NHS Cancer Programme Operations Information. NHS England, 2018. Available from: https://www.england.nhs.uk/wp-content/uploads/2018/04/implementing-timed-prostate-cancer-diagnostic-pathway.pdf. Accessed November 2020.

17. NICE, *Prostate cancer: diagnosis and management NICE guideline [NG131].* 2019. Available from: https://www.nice.org.uk/guidance/ng131. Accessed November 2020.

18. Bjurlin MA, Carroll PR, Eggener S et al. Update of the Standard Operating Procedure on the Use of Multiparametric Magnetic Resonance Imaging for the Diagnosis, Staging and Management of Prostate Cancer. *J Urol.* 2020;203:706-712.

19. Lam TBL, MacLennan S, Willemse PM et al. EAU-EANM-ESTRO-ESUR-SIOG Prostate Cancer Guideline Panel Consensus Statements for Deferred Treatment with Curative Intent for Localised Prostate Cancer from an International Collaborative Study (DETECTIVE Study). *Eur Urol. 2019;* 76:790-813.

INSERT ONTO GP LETTERHEAD

INSET PATIENT DETAILS HERE

Dear insert patient name,

Insert GP organisation name is collaborating with University College London Hospital (UCLH) on a screening study for Prostate Cancer. You have been **randomly** selected from our existing patient database of men between the ages of 50-75 for invitation who may be able to participate. Please be assured no details in relation to you have left the practice.

To participate in the screening study, you would be required to attend a single visit at UCLH where you would be asked to:

Stock photo

1 Provide information on your medical history

Stock photo

2 Provide a blood sample

Stock photo

3 Have a Magnetic resonance imaging (MRI) scan of your prostate

The visit will normally last approximately **1 hour** and mainly occur on Thursdays & Saturdays. Each man attending the study visit will be offered a free return taxi to attend, or reimbursement for other travel arrangements e.g. train, parking costs.

If this is of interest to you, please read the **information sheet** included with this letter.

If after reading the information sheet you want to participate you need to **contact the team at UCLH** via the contact details below. They will be more than happy to discuss any further questions you may have and let you know the next steps should you wish to take part.

Please see the attached safeguarding document in relation to how the study has changed since COVID-19.

Contact the ReIMAGINE study staff on the below details:

Monday – Friday (9am-5pm)
Tel: 078 236 85797 or 0207 679 9280
Email: uclh.reimagine@nhs.net

Saturday
Tel: 078 236 85797
(or email if not concerning a scan on that day)

GP study invitation letter
ReIMAGINE Prostate Cancer Screening, IRAS ID: 251167
Version 2.0, Date 1st July 2020
Page 1 of 1

For peer review only

re|imagine
Prostate Cancer Screening

Forename		Surname			
Date of Birth		Age within range?	☐Yes ☐No	Weight (kg)	
Height (m)		BMI = w/(hxh)			
Address					
GP Name and address				Screening no.	
Contact No.		Email			

Study Eligibility & MRI Safety Questionnaire for PATIENTS (Complete at screening for all respondents)	
**Can I confirm you have never previously been diagnosed with Prostate cancer?** *(Previous diagnosis or treatment are excluded)*	Yes/No
**If eligible are you able to travel to UCLH for the MRI scan? Do you need any special assistance?** *(Men who require assisted living e.g. care home living are excluded)* *Notes:*	Yes/No
**Have you any neurological conditions such as dementia which may limit your ability to participate that we need to know of?** *(Dementia or other neurological condition meaning participant lacks the capacity to consent is an exclusion)*	Yes/No

"The next group of questions will assess whether it is safe for you to have an MRI. Please note they will be repeated on the day by the radiographer performing the scan in line with hospital policy".

1. **Have you ever had a cardiac device, even if it has been removed?** *Eg: pacemaker, implantable cardioverter defibrillator (ICD), cardiac monitor or a REVEAL device?*	Yes/No
2. **Have you ever had an operation to your heart?** *Eg: cardiac stents, heart valves, repair of holes in the heart?*	Yes/No
3. **Have you ever had an operation to your head, neck, spine, eyes or ears?** *Eg: aneurysm clips, coils, shunts, cochlear / inner ear devices, or any other implants in the head*	Yes/No
4. **Have you ever had metal fragments in your eyes or other part of your body?** *E.g. metal filings, shrapnel, bullets, debris etc?*	Yes/No
5. **Have you ever had any type of electronic, mechanical or magnetic implants?** Eg: *neuro-stimulator, tissue expanders, Stanmore non-invasive grower, drug infusion pumps etc?*	Yes/No
6. **Have you ever had any other implants or devices (NOT dental)** *Eg: orthopaedic implants, joint replacements, plates, clips, IVC filters, stents, coils, clamps, access ports, penile implant, sutures, staples, metal or wire mesh, IUD or contraceptive coil, or any other device?*	Yes/No
7. **Have you ever swallowed an endoscopy capsule or any other device?** *Can you confirm whether it is has left the body? Yes/No*	Yes/No
8. **Have you had any operations to any part of your body in the past 3 months?**	Yes/No
9. **Do you wear dentures, a dental plate or a brace?** *(will have to remove if metal and removable)*	Yes/No
10. **Do you have epilepsy, or have you had a seizure in the past?**	Yes/No
11. **Do you have a tattoo/s or permanent makeup?**	Yes/No
12. **Do you wearing coloured contact lens, piercings or hearing aids?** (will have to remove for scan)	Yes/No
13. **Are you currently wearing a transdermal patch (medication/nicotine patch)?**	Yes/No

re|imagine
Prostate Cancer Screening

MRI Checklist for Injections (Complete for every visit)	
**Do you have any allergies? If yes please list**	Yes/No
**Are you taking anticoagulants (warfarin, aspirin, heparin) or any medication that thins your blood?**	Yes/No

1. **Have you ever had Buscopan®? If yes, did you experience any side effects?** *Staff Note: Buscopan is given to stop motion in the bowels which can distort the images. As an over the counter drug this is commonly used to treat stomach cramps.*	Yes/No
2. Do you have tachycardia or any heart problems?	Yes/No
3. Do you have megacolon (swollen large bowel) or narrowing of the bowel?	Yes/No
4. Do you have myasthenia gravis (a disease that causes muscle weakness)?	Yes/No
5. Do you have a difficulty or pain passing urine (particularly men with prostate problems)?	Yes/No
6. Do you have glaucoma or under investigation for glaucoma?	Yes/No

Staff USE ONLY:

If any questions above are answered **'yes'** please follow the below guidance

Question 1: Absolute contraindication to MRI **at UCH**

Question 2, 3, 5 & 6: Information pertaining to the device insitu would be required. **Contact myself, or MRI department to discuss**

Question 4: As per above, metal fragments that have not been removed would exclude the patient. Patients whom are unaware of removal may require an X-ray prior to MRI for confirmation. **Contact myself or MRI department to discuss**

Question 7: Endoscopy capsule must be known to have left the body prior to MRI

Question 8: Where recent surgery has involved device insertion, again we would require further information as per above. **Contact** lucy.caselton@nhs.net **or the MRI department to discuss**

Questions 9-13 and 'Checklist for injection' section wouldn't exclude patients from MRI

re|imagine
Prostate Cancer Screening

COVID-19 questions	
1. Have you tested positive for COVID-19 in the last 10 days? ***NB.*** *If 'no' and symptoms reoccur a repeat test would need to be completed before entry to hospital is permissible.*	Yes/No
2. Are you waiting for a COVID-19 test or the results?	Yes/No
3. Do you have any of the following symptoms: • New, continuous cough* • High temperature or fever • Loss of, or change in, sense of smell or taste? ** A new, continuous cough means coughing for longer than an hour or three or more coughing episodes in 24 hours. If you usually have a cough, it may be worse than usual.*	Yes/No
4. Do you live with someone who has either tested positive for COVID-19 or had symptoms of COVID-19 in the last 14 days? ***N.B.*** *If the answer is yes, a repeat test would need to be completed before entry to hospital is permissible.*	Yes/No

Travel Questions	
1. What would be your preferred method of travel to the hospital? ………………………………………… We can arrange a taxi for you, or reimbursement if you would prefer to arrange this yourself (e.g. walk, bus, own taxi, own car and parking).	
2. If we arrange a taxi for you, do you have access to a facemask or appropriate covering that you can wear during the trip?	Yes/No
3. Are there any special instructions we should provide to the taxi driver in relation to where you would like to be collected from?	

Form Completed by		Date	

*** Please remind the patient that the study visit and access to the scanner area will currently be limited to them only. If they travel with a companion, he/she may have to wait outside the hospital building, unless their assistance is required to travel to the room itself.**

Note to CTP: Please send the document with details in relation to the taxi drop off/pick up area and instructions of how to find the scanner room when in the hospital.

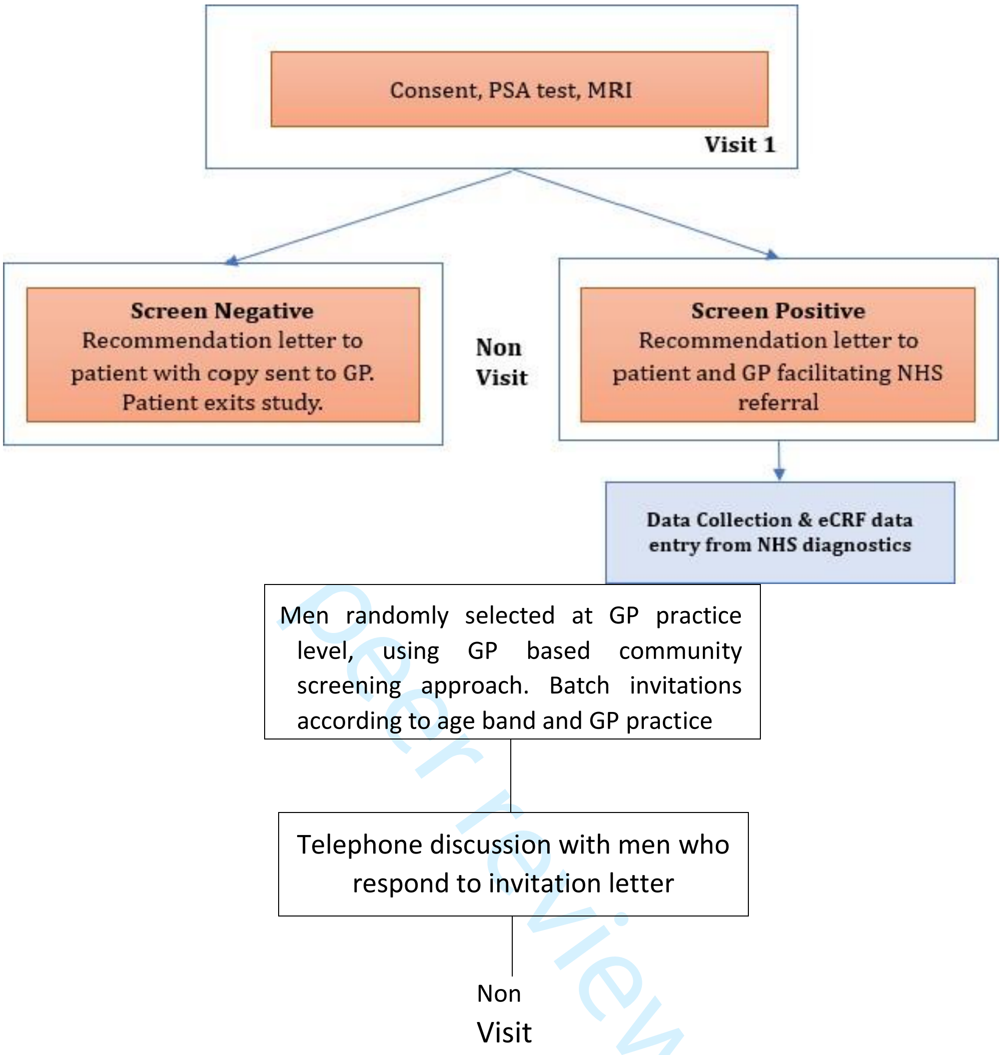
Consent, PSA test, MRI
Visit 1
Screen Negative
Recommendation letter to patient with copy sent to GP. Patient exits study.
Non Visit
Screen Positive
Recommendation letter to patient and GP facilitating NHS referral
Data Collection & eCRF data entry from NHS diagnostics
Men randomly selected at GP practice level, using GP based community screening approach. Batch invitations according to age band and GP practice
Telephone discussion with men who respond to invitation letter
Non
Visit

University College London Hospitals
NHS Foundation Trust

reimagine
Prostate Cancer Screening

**Patient name**	
**Hospital no.**	
**MRI date**	/ /

**REPORTER 2 / 3:**		
MRI scan result	☐ Screen positive	☐ Screen negative
MRI scan quality	☐ Diagnostic	☐ Non-Diagnostic

Comments:

Date & Time of Report:

Please use the following reporting criteria to ascribe screen positive or screen negative status:

Axial T2 (Likert)	Axial b2000 (Likert)	Screen
focal 4-5/5 or diffuse 3/5	focal 4-5/5	Positive
focal 4-5/5	focal 1-2/5 or diffuse 3/5	Negative
diffuse 3/5	1-2/5 or Likert diffuse 3/5	Negative
1-2/5	ANY	Negative
ANY	1-2/5	Negative
Non-diagnostic (artefact)	1-2/5 or diffuse 3/5	Negative
1-2/5 or diffuse 3/5	Non-diagnostic (artefact)	Negative
4-5/5	Non-diagnostic (artefact)	Non-diagnostic (Recall)
Non-diagnostic (artefact)	4-5/5	Non-diagnostic (Recall)
Non-diagnostic (artefact)	Non-diagnostic (artefact)	Non-diagnostic (No recall)

ReIMAGINE PCa Screening (IRAS 251167)
MRI Reporter 2/3 CRF
V1.1, dated 20th Nov 2019

uclh University College Hospital · National Hospital for Neurology and Neurosurgery · Eastman Dental Hospital · Royal National Throat, Nose and Ear Hospital · Royal London Hospital for Integrated Medicine

University College London Hospitals
NHS Foundation Trust

reimagine
Prostate Cancer Screening

**Patient name**	
**Hospital no.**	
**MRI date**	

**MRI REPORT**			
**REPORTER 1:**			
MRI Prostate volume (ml)			
MRI scan result	☐ Screen Positive	☐ Screen Negative	
Lesion laterality	☐ Left	☐ Right	☐ Both
Lesion location 1	☐ TZ	☐ PZ	☐ CZ
Lesion location 2	☐ Base	☐ Mid	☐ Apex
Report comments			
MRI scan quality	☐ Diagnostic	☐ Non - Diagnostic	
Date & Time of Report			

N.B. If there is a visible concordant positive lesion on the MRI (seen on both T2 & b2000) please report as positive on a non-diagnostic scan i.e. the whole prostate is not visible due to artefact(s).

Please use the following reporting criteria to ascribe screen positive or screen negative status:

Axial T2 (Likert)	Axial b2000 (Likert)	Screen
focal 4-5/5 or diffuse 3/5	focal 4-5/5	Positive
focal 4-5/5	focal 1-2/5 or diffuse 3/5	Negative
diffuse 3/5	1-2/5 or Likert diffuse 3/5	Negative
1-2/5	ANY	Negative
ANY	1-2/5	Negative
Non-diagnostic (artefact)	1-2/5 or diffuse 3/5	Negative
1-2/5 or diffuse 3/5	Non-diagnostic (artefact)	Negative
4-5/5	Non-diagnostic (artefact)	Non-diagnostic (Recall)
Non-diagnostic (artefact)	4-5/5	Non-diagnostic (Recall)
Non-diagnostic (artefact)	Non-diagnostic (artefact)	Non-diagnostic (No recall)

ReIMAGINE PCa Screening (IRAS 251167)
MRI Reporter 1 CRF
V1.0, dated 1st November 2019

uclh University College Hospital · National Hospital for Neurology and Neurosurgery · Eastman Dental Hospital · Royal National Throat, Nose and Ear Hospital · Royal London Hospital for Integrated Medicine

Appendix I: Amendments made to the ReIMAGINE Prostate Cancer Screening Study Protocol

Two non-substantial amendments were made to the study protocol during the duration of the study:

Non-substantial amendment 1

The first non-substantial amendment was submitted for review in June 2020. Review by the study sponsor (UCL) determined this was a category C amendment that did not require study wide review, with notification to UK Health Research Authority only. The purpose of the amendment was modification of study documents (Participant Information Sheet, PIS) to navigate participants to the study privacy notice which informed participants about the information being collected and its intended use, a General Data Protection Regulation (GDPR) requirement. At this time, we incorporated minor updates to the study protocol (expansion on what categories of aggregate data we intend to collect from PIC sites / GP practices) and the study consent form (mention of the privacy notice) to correct administrative omissions. The amendment was implemented at site in July 2020.

Non-substantial amendment 2

The second non-substantial amendment followed a temporary closure of recruitment to the study enforced by the study sponsor and NHS site in response to the COVID-19 pandemic (March 2020). The study team consulted with the Patient and Public Involvement (PPI) sub-committee on what modifications we may need to make to documentation or study design so to safely re-open to recruitment and provide assurance to potential new participants that COVID-19 concerns had been considered. This was submitted in July 2020, once again categorised as category C, and implemented in August 2020 when approval to reopen recruitment was granted by the study sponsor.

In response to the COVID-19 pandemic, the PIS was amended to reflect the change in travel allowance/arrangements made by the study team. All participants were offered the option of a private return taxi for their study visit, or reasonable travel allowance greater than the original £30. The study invitation letter (sent by GPs) was also updated to note the same change and navigate men to a new one-page information sheet in relation to study safeguards during the COVID-19 pandemic, the content of which was largely driven by the PPI committee.

For peer review only

Appendix II: MRI protocol

All studies will be performed with the same protocol on a single 3 T scanner (Achieva®, Philips Healthcare, Netherlands) using a 32-channel pelvic-phased array coil and a bi-parametric acquisition protocol. Briefly, sequences will include an axial turbo spin echo and axial diffusion weighted imaging using a high b value at 2000 s mm–2. Exemplar sequence parameters are illustrated in the table below. Total protocol acquisition time <10 minutes.

Clinical bi-parametric MRI at 3T

**Parameter**	**T_2 axial**	**DWI b 2000 s mm^{-2}**
**Repetition time (ms)**	5407	2000
**Echo time (ms)**	100	78
**Flip angle (degree)**	90	90
**Orientation**	Axial	Axial
**Slice thickness (mm)**	3	5
**Matrix size**	300 × 290	168 × 169
**Field of view (mm)**	180	220
**Fat suppression**	No	SPIR
**Time for scan (min)**	05:13	03:40

The above images will be prospectively reported by radiologists to ascribe a positive/negative screen result as described in the ReIMAGINE Prostate Cancer Screening study protocol. Reports will be generated in UCLH EPIC using a study specific reporting template.

At the same sitting, patients will undergo exploratory imaging for acquisition of imaging to derive Luminal Water Fraction (LWF) maps. Imaging will be performed with the same scanner and coil setup. An 8-echo multi-echo spin echo sequence will be employed to provide axial imaging of the prostate. Exemplar sequence parameters are illustrated in the table below. Total exploratory imaging protocol time < 10 minutes.

For peer review only

Exploratory LWF MRI at 3T

**Parameter**	**LWF Imaging**
**Repetition time (ms)**	8956
**Echo time/spacing (ms)**	31.25/31.25
**Flip angle (degree)**	90
**Orientation**	Axial
**Slice thickness (mm)**	4
**Matrix size**	300 × 290
**Field of view (mm)**	180
**Fat suppression**	No
**Time for scan (min)**	05:13

Positive/negative screen status/quantitative analysis and concordance with the bi-parametric MRI will be performed. Reports will not be made available to the clinical team.

For peer review only

Appendix III – The ReIMAGINE Screening Study Group

**Trial Sponsor:**	University College London (UCL)
**Trial Coordination:**	UCL Urology, Research Department of Targeted Intervention, Division of Surgery and Interventional Science
**Funders:**	The Medical Research Council, U.K. (MRC), grant number MR/R014043/1 and Cancer Research U.K. (CRUK)

Trial Management Group

**Caroline M Moore**	Co-Chief Investigator, Urologist - UCLH / UCL
**Mark Emberton**	Co-Chief Investigator, Urologist - UCLH / UCL
**Shonit Punwani**	Co-Investigator, Radiologist - UCLH / UCL
**Neil McCartan**	Clinical Project Management – UCL
**Louise Brown**	Co-Investigator – UCL MRC Clinical Trials Unit
**Mieke Van Hemelrijck**	Co-Investigator, Cancer Epidemiologist - KCL
**Ton Coolen**	Co-Investigator, Applied Mathematics - London Institute for Mathematical Sciences
**Aida Santaollala**	Cancer Epidemiology Group – KCL
**Teresa Marsden**	Clinical Research Fellow, Urology – UCLH / UCL
**Harbir Sidhu**	Consultant Radiologist – UCLH
**Douglas Kopcke**	Honorary Consultant Radiologist - UCLH
**Giorgio Brembilla**	Clinical Research Fellow, Radiology – UCLH / UCL
**Francesco Giganti**	Clinical Research Fellow, Radiology – UCLH / UCL

ReIMAGINE Screening Study Group

**Joanna Hadley**	Clinical Trial Practitioner – UCL
**Fatima Akbar**	Clinical Trial Practitioner - UCL
**Ged Corbett**	Project Manager – UCL TRO
**Manuel Rodriguez-Justo**	Pathologist – UCLH / UCL
**Elizabeth Isaac**	Imaging Trials Unit - UCLH
**Steve Tuck**	ReIMAGINE Consortium Patient Representative
**Saran Green**	Patient and Public Involvement - KCL
**Charlotte Moss**	Data Management - KCL
**Anna J Haire**	Data Management - KCL

For peer review only

**Teresita Beeston**	Imaging Trials Unit - UCLH
**Joey Clement**	Imaging Trials Unit - UCLH
**Fiona Gong**	Centre for Medical Imaging – UCL
**Lucy Casselton**	Centre for Medical Imaging – UCL
**Anna Wingate**	Laboratory Manager – UCL
**Katerina Soteriou**	Imaging Trials Unit - UCLH
**Charlotte Bevan**	Biological Research Committee - ReIMAGINE
**Paul Boutros**	Biological Research Committee - ReIMAGINE
**Andrew Feber**	Biological Research Committee - ReIMAGINE
**Hayley Whitaker**	Biological Research Committee - ReIMAGINE
**Caroline Dive**	Scientific Advisory Board - ReIMAGINE
**Eytan Domany**	Scientific Advisory Board - ReIMAGINE
**Malcolm Mason**	Scientific Advisory Board - ReIMAGINE
**Anwar Padhani**	Scientific Advisory Board - ReIMAGINE
**Eric Aboagye**	Steering Committee - ReIMAGINE
**Richard Kaplan**	Steering Committee - ReIMAGINE
**Chris Parker**	Steering Committee - ReIMAGINE
**Peter Parker**	Steering Committee - ReIMAGINE

ew only

SPIRIT

STANDARD PROTOCOL ITEMS: RECOMMENDATIONS FOR INTERVENTIONAL TRIALS

SPIRIT 2013 Checklist: Recommended items to address in a clinical trial protocol and related documents*

Section/item	Item No	Description	Page no
**Administrative information**			
Title	1	Descriptive title identifying the study design, population, interventions, and, if applicable, trial acronym	**1**
Trial registration	2a	Trial identifier and registry name. If not yet registered, name of intended registry	**6**
	2b	All items from the World Health Organization Trial Registration Data Set	**6**
Protocol version	3	Date and version identifier	**6**
Funding	4	Sources and types of financial, material, and other support	**6**
Roles and responsibilities	5a	Names, affiliations, and roles of protocol contributors	**1**
	5b	Name and contact information for the trial sponsor	**6**
	5c	Role of study sponsor and funders, if any, in study design; collection, management, analysis, and interpretation of data; writing of the report; and the decision to submit the report for publication, including whether they will have ultimate authority over any of these activities	**6**
	5d	Composition, roles, and responsibilities of the coordinating centre, steering committee, endpoint adjudication committee, data management team, and other individuals or groups overseeing the trial, if applicable (see Item 21a for data monitoring committee)	**6**
**Introduction**			
Background and rationale	6a	Description of research question and justification for undertaking the trial, including summary of relevant studies (published and unpublished) examining benefits and harms for each intervention	**1, 2**
	6b	Explanation for choice of comparators	**1, 2**
Objectives	7	Specific objectives or hypotheses	**7**

For peer review only - http://bmjopen.bmj.com/site/about/guidelines.xhtml

Trial design	8	Description of trial design including type of trial (eg, parallel group, crossover, factorial, single group), allocation ratio, and framework (eg, superiority, equivalence, noninferiority, exploratory)	**6, 16**
**Methods: Participants, interventions, and outcomes**			
Study setting	9	Description of study settings (eg, community clinic, academic hospital) and list of countries where data will be collected. Reference to where list of study sites can be obtained	**6**
Eligibility criteria	10	Inclusion and exclusion criteria for participants. If applicable, eligibility criteria for study centres and individuals who will perform the interventions (eg, surgeons, psychotherapists)	**7**
Interventions	11a	Interventions for each group with sufficient detail to allow replication, including how and when they will be administered	**9 - 15**
	11b	Criteria for discontinuing or modifying allocated interventions for a given trial participant (eg, drug dose change in response to harms, participant request, or improving/worsening disease)	**8 - 15**
	11c	Strategies to improve adherence to intervention protocols, and any procedures for monitoring adherence (eg, drug tablet return, laboratory tests)	**8 - 15**
	11d	Relevant concomitant care and interventions that are permitted or prohibited during the trial	**13 - 15**
Outcomes	12	Primary, secondary, and other outcomes, including the specific measurement variable (eg, systolic blood pressure), analysis metric (eg, change from baseline, final value, time to event), method of aggregation (eg, median, proportion), and time point for each outcome. Explanation of the clinical relevance of chosen efficacy and harm outcomes is strongly recommended	**7**
Participant timeline	13	Time schedule of enrolment, interventions (including any run-ins and washouts), assessments, and visits for participants. A schematic diagram is highly recommended (see Figure)	**9 - 15**
Sample size	14	Estimated number of participants needed to achieve study objectives and how it was determined, including clinical and statistical assumptions supporting any sample size calculations	**16**
Recruitment	15	Strategies for achieving adequate participant enrolment to reach target sample size	**9, 18**
**Methods: Assignment of interventions (for controlled trials)**			
Allocation:			

Sequence generation	16a	Method of generating the allocation sequence (eg, computer-generated random numbers), and list of any factors for stratification. To reduce predictability of a random sequence, details of any planned restriction (eg, blocking) should be provided in a separate document that is unavailable to those who enrol participants or assign interventions	**9**
Allocation concealment mechanism	16b	Mechanism of implementing the allocation sequence (eg, central telephone; sequentially numbered, opaque, sealed envelopes), describing any steps to conceal the sequence until interventions are assigned	**9**
Implementation	16c	Who will generate the allocation sequence, who will enrol participants, and who will assign participants to interventions	**9 - 10**
Blinding (masking)	17a	Who will be blinded after assignment to interventions (eg, trial participants, care providers, outcome assessors, data analysts), and how	**12 - 13**
	17b	If blinded, circumstances under which unblinding is permissible, and procedure for revealing a participant's allocated intervention during the trial	**NA**
**Methods: Data collection, management, and analysis**			
Data collection methods	18a	Plans for assessment and collection of outcome, baseline, and other trial data, including any related processes to promote data quality (eg, duplicate measurements, training of assessors) and a description of study instruments (eg, questionnaires, laboratory tests) along with their reliability and validity, if known. Reference to where data collection forms can be found, if not in the protocol	**10 - 15**
	18b	Plans to promote participant retention and complete follow-up, including list of any outcome data to be collected for participants who discontinue or deviate from intervention protocols	**13 - 15**
Data management	19	Plans for data entry, coding, security, and storage, including any related processes to promote data quality (eg, double data entry; range checks for data values). Reference to where details of data management procedures can be found, if not in the protocol	**12 - 15**
Statistical methods	20a	Statistical methods for analysing primary and secondary outcomes. Reference to where other details of the statistical analysis plan can be found, if not in the protocol	**16**
	20b	Methods for any additional analyses (eg, subgroup and adjusted analyses)	**16**

For peer review only - http://bmjopen.bmj.com/site/about/guidelines.xhtml

	20c	Definition of analysis population relating to protocol non-adherence (eg, as randomised analysis), and any statistical methods to handle missing data (eg, multiple imputation)	**16**
**Methods: Monitoring**			
Data monitoring	21a	Composition of data monitoring committee (DMC); summary of its role and reporting structure; statement of whether it is independent from the sponsor and competing interests; and reference to where further details about its charter can be found, if not in the protocol. Alternatively, an explanation of why a DMC is not needed	**Appx**
	21b	Description of any interim analyses and stopping guidelines, including who will have access to these interim results and make the final decision to terminate the trial	**NA**
Harms	22	Plans for collecting, assessing, reporting, and managing solicited and spontaneously reported adverse events and other unintended effects of trial interventions or trial conduct	**13**
Auditing	23	Frequency and procedures for auditing trial conduct, if any, and whether the process will be independent from investigators and the sponsor	**13**
**Ethics and dissemination**			
Research ethics approval	24	Plans for seeking research ethics committee/institutional review board (REC/IRB) approval	**6**
Protocol amendments	25	Plans for communicating important protocol modifications (eg, changes to eligibility criteria, outcomes, analyses) to relevant parties (eg, investigators, REC/IRBs, trial participants, trial registries, journals, regulators)	**6, 15, Appx**
Consent or assent	26a	Who will obtain informed consent or assent from potential trial participants or authorised surrogates, and how (see Item 32)	**10**
	26b	Additional consent provisions for collection and use of participant data and biological specimens in ancillary studies, if applicable	**10**
Confidentiality	27	How personal information about potential and enrolled participants will be collected, shared, and maintained in order to protect confidentiality before, during, and after the trial	**13, 16**
Declaration of interests	28	Financial and other competing interests for principal investigators for the overall trial and each study site	**6, 22**
Access to data	29	Statement of who will have access to the final trial dataset, and disclosure of contractual agreements that limit such access for investigators	**Data statement**

review only

Ancillary and post-trial care	30	Provisions, if any, for ancillary and post-trial care, and for compensation to those who suffer harm from trial participation	**14**
Dissemination policy	31a	Plans for investigators and sponsor to communicate trial results to participants, healthcare professionals, the public, and other relevant groups (eg, via publication, reporting in results databases, or other data sharing arrangements), including any publication restrictions	**3, 14, 17**
	31b	Authorship eligibility guidelines and any intended use of professional writers	**NA**
	31c	Plans, if any, for granting public access to the full protocol, participant-level dataset, and statistical code	**NA**
**Appendices**			
Informed consent materials	32	Model consent form and other related documentation given to participants and authorised surrogates	**Appx**
Biological specimens	33	Plans for collection, laboratory evaluation, and storage of biological specimens for genetic or molecular analysis in the current trial and for future use in ancillary studies, if applicable	**NA**

*It is strongly recommended that this checklist be read in conjunction with the SPIRIT 2013 Explanation & Elaboration for important clarification on the items. Amendments to the protocol should be tracked and dated. The SPIRIT checklist is copyrighted by the SPIRIT Group under the Creative Commons "Attribution-NonCommercial-NoDerivs 3.0 Unported" license.

For peer review only
